# Characterization of pancreatic glucagon-producing tumors and pituitary gland tumors in transgenic mice overexpressing *MYCN* in *hGFAP*-positive cells

**DOI:** 10.18632/oncotarget.12766

**Published:** 2016-10-19

**Authors:** Kathrin Fielitz, Kristina Althoff, Katleen De Preter, Julie Nonnekens, Jasmin Ohli, Sandra Elges, Wolfgang Hartmann, Günter Klöppel, Thomas Knösel, Marc Schulte, Ludger Klein-Hitpass, Daniela Beisser, Henning Reis, Annette Eyking, Elke Cario, Johannes H. Schulte, Alexander Schramm, Ulrich Schüller

**Affiliations:** ^1^ Department of Pediatric Oncology and Hematology, University Children's Hospital Essen, University of Duisburg-Essen, Essen, Germany; ^2^ Centre for Medical Genetics, Ghent University Hospital, Ghent, Belgium; ^3^ Genetics and Nuclear Medicine, Erasmus Medical Center, Rotterdam, The Netherlands; ^4^ Center for Neuropathology, Ludwig-Maximilians University, Munich, Germany; ^5^ Department of Pathology, University Hospital, Münster, Germany; ^6^ Department of Pathology, Technical University, Munich, Germany; ^7^ Department of Pathology, Ludwig-Maximilians University, Munich, Germany; ^8^ Cell Biology, University Hospital Essen, University of Duisburg-Essen, Essen, Germany; ^9^ Genome Informatics, University Hospital Essen, University of Duisburg-Essen, Essen, Germany; ^10^ Department of Pathology, University Hospital Essen, University of Duisburg-Essen, Essen, Germany; ^11^ Division of Gastroenterology and Hepatology, University Hospital Essen, University of Duisburg-Essen, Essen, Germany; ^12^ Pediatric Oncology and Hematology, Charité University Medicine, Berlin, Germany; ^13^ Institute of Neuropathology, University Medical Center, Hamburg-Eppendorf, Hamburg, Germany; ^14^ Research Institute Childrens Cancer Center, Hamburg, Germany; ^15^ Department of Pediatric Oncology and Hematology, University Medical Center, Hamburg-Eppendorf, Hamburg, Germany

**Keywords:** glucagonoma, pancreatic neuroendocrine tumors, MYCN

## Abstract

Amplification or overexpression of *MYCN* is involved in development and maintenance of multiple malignancies. A subset of these tumors originates from neural precursors, including the most aggressive forms of the childhood tumors, neuroblastoma and medulloblastoma. In order to model the spectrum of *MYCN*-driven neoplasms in mice, we transgenically overexpressed MYCN under the control of the human GFAP-promoter that, among other targets, drives expression in neural progenitor cells. However, LSL-MYCN;hGFAP-Cre double transgenic mice did neither develop neural crest tumors nor tumors of the central nervous system, but presented with neuroendocrine tumors of the pancreas and, less frequently, the pituitary gland. Pituitary tumors expressed chromogranin A and closely resembled human pituitary adenomas. Pancreatic tumors strongly produced and secreted glucagon, suggesting that they derived from glucagon- and GFAP-positive islet cells. Interestingly, 3 out of 9 human pancreatic neuroendocrine tumors expressed *MYCN,* supporting the similarity of the mouse tumors to the human system. Serial transplantations of mouse tumor cells into immunocompromised mice confirmed their fully transformed phenotype. MYCN-directed treatment by AuroraA- or Brd4-inhibitors resulted in significantly decreased cell proliferation *in vitro* and reduced tumor growth *in vivo*. In summary, we provide a novel mouse model for neuroendocrine tumors of the pancreas and pituitary gland that is dependent on MYCN expression and that may help to evaluate MYCN-directed therapies.

## INTRODUCTION

MYC-family proteins are universal amplifiers of gene expression in normal cells [1], but display specific activity in tumor cells, thus regulating their proliferation, apoptosis, growth and differentiation [2]. Ever since the identification of *MYCN* amplification in primary neuroblastoma [3], an oncogenic role of MYCN has been assumed. MYCN is able to promote proliferation and repress differentiation, which promotes tumor growth in numerous malignancies. Under physiological conditions, expression of MYCN is tightly controlled and subject to rapid turnover due to phosphorylation-dependent proteasomal degradation in part controlled by Aurora A kinase [4]. An enhanced gene dosage due to amplification of the associated genomic region is observed in about 20% of neuroblastoma, giving rise to the most aggressive subtype of the disease (reviewed in [5]). Targeted overexpression of MYCN in the neural crest, which has been shown in different model systems (termed TH-MYCN and LSL-MYCN;DBH-iCre, respectively) [6] [7], results in neuroblastic tumors closely resembling the respective human malignancies. Similarly, overexpression of mutationally stabilized MYCN in progenitor cells of the mouse cerebellum generates MYCN-driven medulloblastomas [8]. MYCN is also deregulated in many tumors of neuroectodermal origin [9]. In pancreatic neuroendocrine tumors (PanNET) the role of MYC proteins is largely unknown, although c-MYC has been suggested to act as an oncogene in a fraction of acinar cell carcinomas [10] and ductal adenocarcinomas [11]. PanNETs, which synthesize and secrete hormones, are hormonally syndromic or non-syndromic. Those that are syndromic may produce clinical conditions, such as the insulinoma syndrome, gastrinoma syndrome (also known as Zollinger-Ellison syndrome), glucagonoma syndrome and VIPoma syndrome (also known as Verner-Morrison syndrome, ordered by declining frequency, for review see [12],[13]). Until recently, the existing mouse models for glucagonoma were established by conditional deletion of tumor-suppressor genes including MEN1 or by combined loss of p53 and Rb [14,15]. These model systems used insulin-promoter or renin-promoter driven expression of Cre recombinase, respectively, to induce target gene deletion. However, no driving oncogene such as MYCN has been linked to tumor formation in these mice.

For a long time, MYCN was considered as a poor therapeutic target. However, development of inhibitors targeting Brd4 (bromodomain containing 4) and AuroraA kinase paved new avenues for interference with MYCN function [16,17]. JQ1 abrogates Brd4-functions and inhibited tumor progression in xenograft models [18] as well as in the recently developed LSL-MYCN;DBH-iCre neuroblastoma mouse model [7]. MLN 8237, which promotes MYCN degradation by AuroraA functions, prolongs the survival of mice in the TH-MYCN model of neuroblastoma [17] and repressed cell viability of neuroblastoma cells *in vitro* [7].

In this study we present a novel mouse model for MYCN induced tumors by targeting MYCN-expression to hGFAP-positive cells. GFAP is a protein widely expressed in the developing nervous system as well as in neuroendocrine cells in adulthood. We characterize the MYCN induced neuroendocrine tumors in mice and provide insights into their biology. We further demonstrate that newly established stable cell lines and xenografts derived from primary mouse tumors are excellent new models to evaluate MYCN-targeted therapies.

## RESULTS

### LSL-MYCN;hGFAP-Cre mice develop abdominal and pituitary tumors

**Figure 1:**
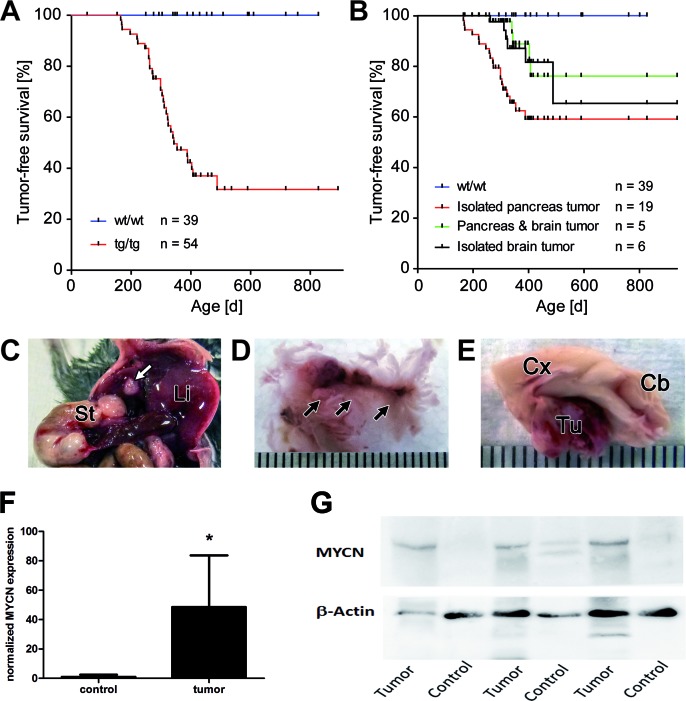
Tumor development in LSL-MYCN;hGFAP-Cre double transgenic mice **A.** Kaplan-Meier analyses of transgenic (tg/tg) tumor bearing mice compared with their littermate wild type controls (wt/wt). Tumor formation was detectable only in mice at an age of 180 days and older (30/54 = 56%). **B.** Kaplan-Meier analyses of tumor bearing mice depending on tumor localization. The curves represent the survival in groups of animals with isolated pancreatic or head tumors or with tumors in both localizations in the same individual. No significant differences in the onset of tumor development were observed as a function of tumor localization. **C.** Macroscopic examination revealed small nodules in the pancreas (white arrow), St: stomach, Li: liver. **D.** Nodule-like tumors in the pancreas are marked by arrows. **E.** Macroscopic appearance of a mouse brain with a tumor (Tu) adjacent to the pituitary gland. Cb: cerebellum, Cx: cortex. Bars, C-E: 0.1 cm. **F.** qRT-PCR reveals low expression of MYCN in control tissue and significantly higher expression of MYCN in tumors (*p* < 0.05). **G.** Western Blot analysis confirms higher MYCN expression in tumors compared with normal control tissue (i.e. tumor-free brain of the same mouse).

Double transgenic LSL-MYCN;hGFAP-Cre mice developed malignancies with an incidence of 59% (32/54), while none of the control single transgenic animals developed tumors (*p* < 0.0001 for tumor formation, Figure 1A). The vast majority of tumors arose between 6 months and 11 months of age (Figure 1A). Tumors were localized either in the abdomen (*n* = 20) or in the head (*n* = 7). Five mice presented with tumors at both sites, but tumor onset did not significantly differ between localizations (Figure 1B). Abdominal tumors were found in the pancreas, with involvement of the liver observed in four animals (Figure 1C, 1D). Head tumors were located at the site of the pituitary gland (Figure 1E). Removal of the loxP-cassette was detectable in all of the GFAP-expressing tissues (not shown), and this was confirmed by *in vivo* bioluminescence imaging (Supplemental Figure 1). Tumors had significantly elevated MYCN mRNA levels compared to controls (Figure 1F) and that also held true for MYCN protein expression (Figure 1G). Thus, targeted MYCN expression in hGFAP-expressing cells of transgenic mice results in tumor formation in pancreatic and pituitary tissue.

### Pancreatic tumors as well as pituitary tumors in LSL-MYCN;hGFAP-Cre mice are of neuroendocrine origin

**Figure 2:**
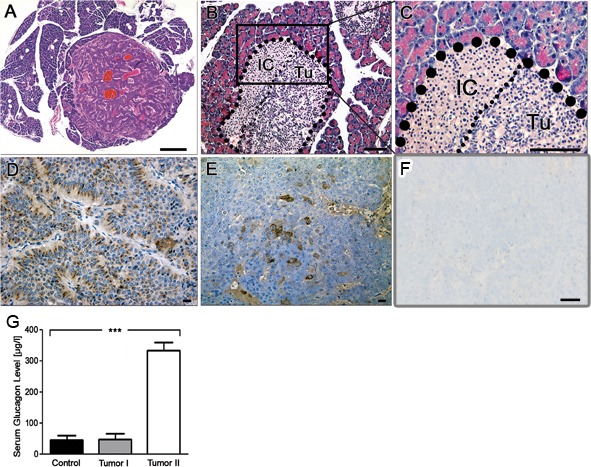
Immunohistochemistry reveals neuroendocrine origin of tumors in LSL-MYCN;hGFAP-Cre mice **A.** H&E staining of a pancreatic tumor with neuroendocrine features. Bar, 200 μm. **B.**, **C.** Higher magnification shows close-up reveals localization of the tumor to the site of pancreatic cells. IC: islet cells, Tu: tumor cells. **D.** Chromogranin A staining confirmed the diagnosis of a neuroendocrine tumor. **E.** Glucagon staining of pancreatic tumors arising in LSL-MYCN;hGFAP-Cre transgenic mice. **F.** Absence of insulin staining in pancreatic tumors. Bars in B-F, 50 μm. **G.** Glucagon levels in blood plasma of control animals or tumor-bearing LSL-MYCN;hGFAP-Cre mice.

**Figure 3:**
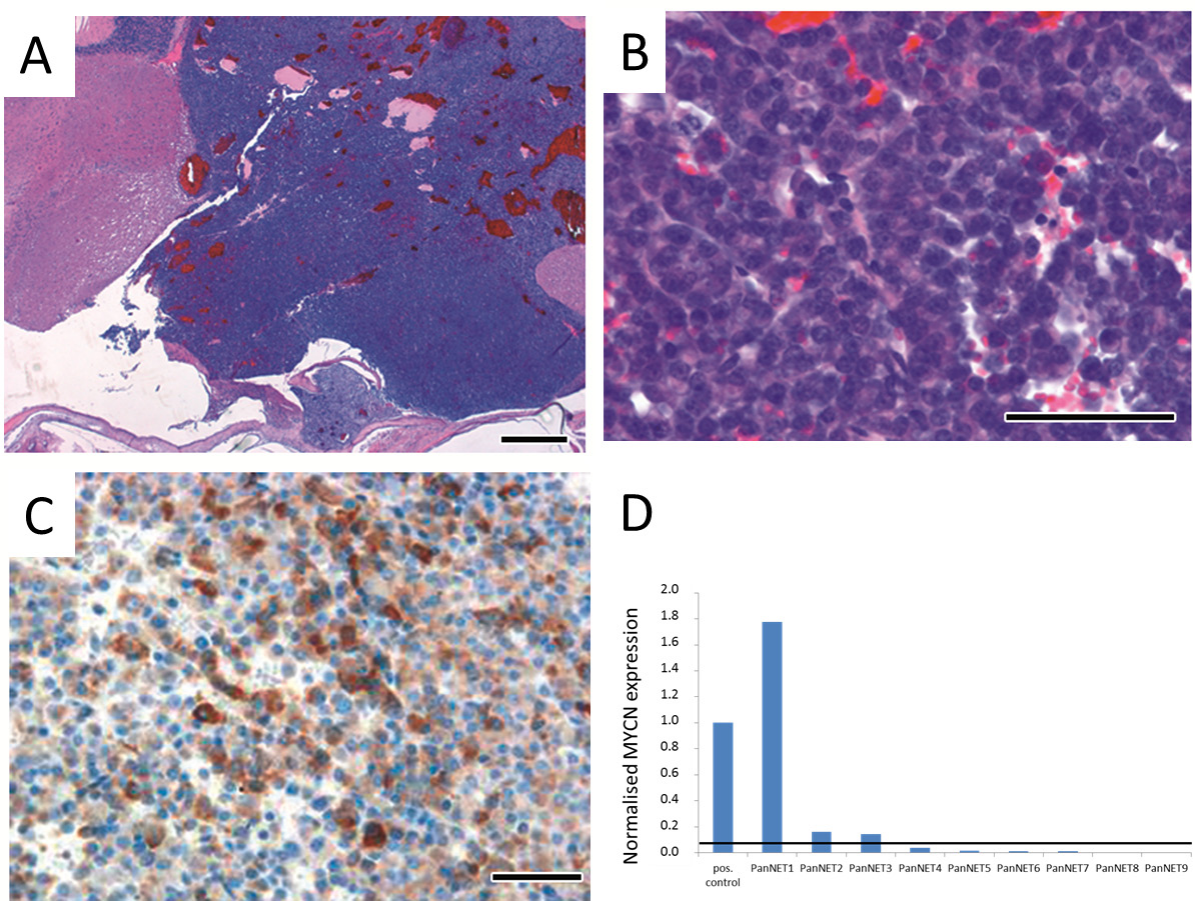
Immunohistochemistry of brain tumors in LSL-MYCN;hGFAP-Cre mice confirmed their neuroendocrine origin **A.**, **B.** H&E stained sections of tumors arising in the pituitary gland at lower and higher magnification, respectively. **C.** Positive chromogranin A immunostaining of a pituitary tumor. Bars, 50 μm. **D.** Normalized *MYCN* mRNA expression in a series of nine human PanNETs. *MYCN* expression was normalized to the expression of β-Actin and this value was set to “1” in the positive control, a human neuroblastoma cell line with high *MYCN* levels due to an amplification of the *MYCN* oncogene. The horizontal line depicts a threshold of 5% of *MYCN* expression in the positive control.

Hematoxylin and eosin (H&E) stained sections of the pancreas showed well-demarcated tumor nodules of varying size that resembled human neuroendocrine tumors (Figure 2A). Small tumors appeared to arise from pancreatic islet cells (Figure 2B and 2C). Proliferation of tumor cells was moderate as reflected by a low percentage of Ki-67 positive cells (data not shown). Tumor cells were positive for chromogranin A (Figure 2D) and glucagon (Figure 2E), while insulin staining was negative (Figure 2F). The corresponding mice were found to have elevated glucagon serum levels (Figure 2G), while insulin levels were in physiological range (data not shown). H&E stained sections of the pituitary tumors revealed similarities to human pituitary adenomas arising in the anterior part of the pituitary gland (Figure 3A-3B). As in the pancreas, pituitary tumors were positive for chromogranin A (Figure 3C). We next analysed the *MYCN* status in a series of nine histologically validated human PanNETs to check whether this oncogene is also expressed in the human disease. Here, comparison of tumor and surrounding normal tissue verified *MYCN*, but not *c-MYC* mRNA expression in three of the nine tumors analysed (Figure 3D), while neither *MYCN* nor c-*MYC* amplifications were detectable. These findings indicate that targeting *MYCN* expression to GFAP-positive cells cause glucagon-producing pancreatic neuroendocrine tumors and pituitary adenomas in transgenic mice and that *MYCN* expression is detectable in a subset of human PanNETs.

### Transcriptomes of murine MYCN-driven neuroendocrine tumors show patterns of canonical MYC-related mRNA signatures

**Figure 4:**
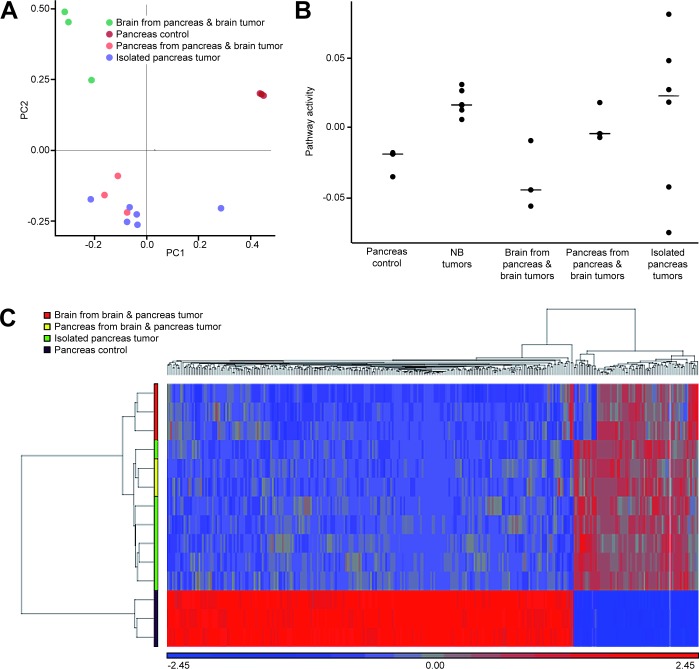
Microarray-derived mRNA profiles obtained from tumors of LSL-MYCN;hGFAP-Cre mice were compared to control pancreata **A.** Principal component analyses (PCA) revealed that all tumors group according to the site of tumor origin as indicated and that these tumor profiles cluster apart from the control profiles (“Pancreas control”). **B.** MYCN pathway activity scores were calculated for all LSL-MYCN;hGFAP-Cre tumor profiles and compared to profiles of MYCN-driven neuroblastoma. **C.** Hierarchical clustering of genes discriminating pancreatic LSL-MYCN;hGFAP-Cre tumors from normal pancreas reveals tumor-specific patterns independent of the tumor site origin.

Next, we wanted to identify disease associated transcriptional patterns by comparing mRNA profiles obtained from normal murine pancreas [19] with those from pancreatic and pituitary tumors of LSL-MYCN;hGFAP-Cre mice. Using principle component analyses, we were able to separate expression patterns of pancreatic tumors from pituitary tumors and from normal pancreatic control tissue (Figure 4A). Moreover, when mRNA profiles from murine MYCN-driven neuroblastoma were included [7], all tumors arising in LSL-MYCN;hGFAP-Cre mice clustered together, while MYCN-induced neuroblastomas were grouped separately (PCA2, Supplemental Figure 2). We therefore compared the specific *MYCN* pathway activity score in the mRNA profiles of all tumors. This revealed a specific *MYCN*-signature described for MYCN-driven murine neuroblastomas also in the pancreatic tumors of LSL-MYCN;hGFAP-Cre mice (Figure 4B). Furthermore, down-regulated mRNAs of the kallikrein peptidase family (*Klk1, Klk1b3, Klk1b5*) and the cholecystokinin receptor type A (*CCKAR*), and expression of *cdh2* coding for N-cadherin as well as somatostatin (Supplemental Table 1) discriminated pancreatic tumors from pancreatic controls. Hierarchical clustering of the differentially expressed genes then identified a common pattern of gene regulation in all tumors arising in the MYCN-GFAP model (Figure 4C). Thus, mRNA profiling allowed both, the recognition of tumor-specific and *MYCN*-specific signatures in LSL-MYCN;hGFAP-Cre induced tumors.

### Stable cell lines derived from LSL-MYCN;hGFAP-Cre induced pancreatic tumors are susceptible to MYCN-directed small molecule inhibition *in vitro* and *in vivo*

**Figure 5:**
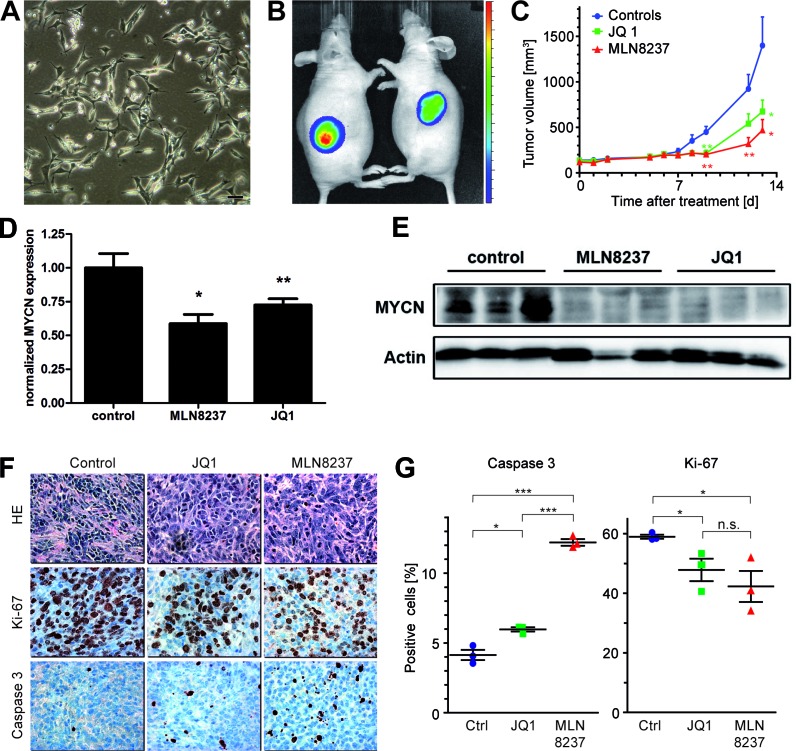
**A.** Morphology of a cell line, designated Pank4, which was derived from a pancreatic tumor of a LSL-MYCN;hGFAP-Cre mouse. Bar, 200 μm. **B.** Xenografts of Pank4 are luciferase-positive in immunocompromised mice. **C.** Growth curves of xenograft tumors, induced by grafting Pank4 cells into immunocompromised mice. Mice were treated either with JQ1, MLN8237 or vehicle. **D.** Mice bearing Pank4-induced xenografts were subjected to forced treatment over three days with either with JQ1, MLN8237 or vehicle (control, “*”: *p* < 0.05; “**”: *p* < 0.01). Hereafter, tumors were checked for MYCN mRNA expression and **E.** MYCN protein expression. **F.** Histology of tumors analysed in C. Tumors were stained with H&E, Ki-67 or an antibody detecting cleaved caspase 3. **G.** Statistical evaluation of differentially treated tumors with respect to the fraction of tumor cells staining positive for cleaved Caspase-3 or Ki-67, respectively.

To establish *in vitro* systems for functional characterization of our newly developed tumor model, cells from a pancreatic tumor of a LSL-MYCN;hGFAP-Cre mouse were isolated and cloned to obtain stable cell lines. One of these cell lines, designated Pank4, displayed undifferentiated morphology (Figure 5A) and provided a positive luciferase signal in bioluminescence imaging (Supplemental Figure 3), indicating activation of the transgenic cassette. Pank4 cells were able to grow as xenografts in immunocompromised mice. *In vivo* luciferase imaging again indicated expression of the transgenic cassette (Figure 5B), which was corroborated by detection of the Cre-mediated removal of the floxed stop sequence (Supplemental Figure 4). To confirm that growth of these tumors was still dependent on the expression of the transgenic driver gene, *MYCN*, animals were treated with a Brd4-inhibitor, JQ1, or an Aurora kinase inhibitor, MLN 8237, once that Pank4-induced xenografts reached a size of 100 mm^3^. Tumor growth in JQ1 and MLN 8237 treated animals was significantly reduced compared to DMSO controls (*p* = 0.05 for treatment with JQ1, and *p* = 0.018 for treatment with MLN 8237, Figure 5C). To analyse on-target effects of both treatment modalities, Pank4-induced xenografts were treated over a period of three days. After that period, tumors were excised and checked for MYCN mRNA and protein expression, respectively (Figure 5D-5E). Here, significant reduction of MYCN mRNA was observed by both treatments. Additionally, MYCN protein levels were reduced by both MLN8237 and JQ1. Histological analyses revealed significantly less proliferating, Ki-67 positive, tumor cells upon targeted treatment (*p* = 0.04 and *p* = 0.036 for treatment with JQ1 or MLN 8237, respectively). Moreover, a significant increase in the fraction of apoptotic cells was observed for animals treated with JQ1 (*p* = 0.012) or with MLN 8237 (*p* < 0.0001, Figure 5D-5E). Thus, targeted treatment of xenografts derived from LSL-MYCN;hGFAP-Cre induced tumors is feasible and significantly delays tumor cell proliferation *in vitro* and *in vivo*.

## DISCUSSION

The MYC family of transcription factors consists of three well characterized members, c-MYC, L-MYC, and MYCN. Deregulation of MYC family members is often associated with aggressive tumor behavior and therapy resistance, and MYC overexpression in amplified tumors represents a therapeutic challenge in many malignancies including neuroblastoma [20]. We here describe a novel model for MYCN-driven tumors that are induced by targeting MYCN to GFAP-positive cells in transgenic mice. These tumors were of neuroendocrine origin and were located either in the pancreas, the pituitary gland or both (Figure 1C). The neuroendocrine nature of the tumors was revealed by immunostaining for the neuroendocrine marker chromogranin A (Figure 2D) and confirmed by global expression profiling. The incidence of tumor formation in our model was considerably lower than in other hGFAP-Cre driven transgenic models, in which penetrance was 100% (hGFAP-Cre x LSL-SmoM2; Schüller et al., Cancer Cell 2008). This suggests that, in general, Cre-mediated excision should be sufficient to achieve a tumor penetrance of 100%, but also that MYCN expression may be variable and that a threshold sufficient for tumor development may not be reached in tumor-free LSL-MYCN;hGFAP-Cre mice. This is supported by the finding that MYCN levels are undetectable or low in target organs in tumor-free animals (Figure 1F-1G). Alternatively, additional hits that have not occurred in tumor-free mice may be required for tumor development. The latter is corroborated by the long latency period until tumor formation becomes evident (> 6 months). However, a potential “second hit” could not be identified yet.

It has been reported that α-cells of the Langerhans islets express GFAP [21,22]. As these α-cells are the main source of glucagon and tumor-bearing LSL-MYCN;hGFAP-Cre mice displayed with elevated blood glucagon levels as well as Glucagon-positive tumors, these findings are in line with the diagnosis of a glucagon-producing PanNET. Expression profiling of tumors confirmed marker expression of neuroendocrine tumors and revealed for the first time a comprehensive view on mRNA patterns in those tumors. Moreover, the role of MYC proteins as transcriptional amplifiers has been suggested by studies demonstrating that MYC overexpression increases the total amount of RNAs both in tumor cells as well as in physiological settings [1,23]. Later, it has been described that oncogenic MYCN acts more specific in shaping the transcriptional profile [2]. This is supposed to explain the emergence of pathway specific signatures related to MYCN by a secondary RNA amplification model ([24], reviewed in [25]). However, small sample size of our dataset does not allow discriminating between MYCN-induced general transcriptional amplification and enrichment of MYCN-specific functional target gene clusters.

In humans, most glucagon-producing PanNETs occur sporadically (87%) and only rarely as a component of the multiple endocrine neoplasia syndrome type 1 (MEN1). In a series of human tumors (*n* = 9) we here showed that *MYCN* is expressed on mRNA level in a subset of these tumors (Figure 3D), while no *MYCN* or *c-MYC* amplifications were detected by FISH analyses (data not shown). Of the glucagon-producing PanNETs, only a fraction is associated with a hormonal syndrome and these are called glucagonomas. They have an incidence of 1 in 20 million [26] with an average age at diagnosis of 52.5 years [27]. Almost all glucagonomas and glucagon-producing PanNETs arise in the pancreas (97%) with only a few tumors being localized extrapancreatically in kidney, duodenum or liver. Most patients with a glucagonoma syndrome develop a so called “necrolytic migratory erythema”, diarrhea, weight loss, diabetes and thromboembolic problems [28], [26,29]. It is of interest to note that mice frequently presented with ruffled fur during onset of tumor formation, which might have had its cause in skin irritations comparable to necrolytic migratory erythema characterizing human glucagonoma patients [28].

The combined appearance of pituitary tumors together with glucagon producing PanNETs is reminiscent of a MEN1 syndrome, in which PanNETs commonly go together with parathyroid and pituitary tumors [30]. However, our LSL-MYCN;hGFAP-Cre double transgenic animals did not develop parathyroid tumors. Moreover, array-CGH analyses of tumors in transgenic mice did not suggest involvement of the *MEN1* locus on mouse chromosome 19, which is syntenic to human chromosome 11q13. Additionally, *MEN1* gene expression was not down-regulated in tumors, rendering mutational inactivation of the *MEN1* gene unlikely. Finally, it remains to be determined, if the spectrum of the human MEN1-syndrome can be fully recapitulated in mice, since glucagon cell specific deletion of MEN1 in mice resulted in the development of insulinomas [31] and not glucagon producing cells. The only other MYCN-driven animal model of MYCN-induced PanNETs was described in zebrafish, yet the developing tumors were similar to human insulinomas [32]. PanNETs expressing glucagon have also been described in mice with a deletion of the glucagon receptor [33], or with a conditional deletion of p53 and Rb [15].

Regarding the question as to whether the presented mouse model can be used for the evaluation of new MYCN-directed therapies, it is of note that transcriptome analyses of the MYCN-GFAP induced tumors revealed a previously described MYCN-related expression signature (Figure 4) [7]. This suggested that MYCN governs the overall expression profile and may function as an oncogenic driver in our mouse model. As MYC proteins themselves are hardly druggable, many efforts have been put into the identification of downstream and upstream regulators of MYC family members. Functional screens have identified Brd proteins, most prominently Brd4, and Aurora kinase A as important check points of MYCN transcription and protein turnover, respectively [16,34]. Therefore, we checked, whether tumor growth depended on MYCN function by JQ1 and MLN8237 in xenografts and in cell lines. For this purpose, stable cell lines were established from pancreatic tumors of LSL-MYCN;hGFAP-Cre mice. Although there is a late onset of primary tumor development in this model (Figure 1A), and tumor growth appears to be slow, tumor-derived cell lines proliferate quickly both *in vitro* and as xenografts in immunocompromised mice (Figure 5A, 5B). Both, cell lines and xenografts responded to JQ1 and MLN8237 treatment and present with reduced MYCN levels on mRNA and protein levels at the end of the therapy. JQ1 down-regulates Brd4-triggered expression of MYCN target genes [18] and this due, at least in part, to interference with Brd4-binding to super-enhancer modules [35]. As MYCN is expressed in our model from a transgenic cassette lacking the super-enhancer module present in the MYCN genomic region, we assume an indirect effect of JQ1 on MYCN expression levels. This could be caused by general reduction of transcription in regressing tumors induced by JQ1. In xenografts, an increased apoptosis rate and decreased proliferation were found (Figure 5F-5G), which is in line with reports on other solid tumors including neuroblastoma [7] and JQ1-treated experimental medulloblastoma [36]. These findings indicate that MYC proteins are required for both tumor initiation and maintenance of the malignant phenotype and corroborate the notion that indirect inhibition of MYCN by targeting Brd-proteins or Aurora A is a promising therapeutic strategy in MYCN-dependent tumors across entities. However, MYC proteins themselves may modulate resistance to JQ1, which has been recently described for pancreatic cancer cells up-regulating c-MYC in response to JQ1 [37]. Thus, we here not only describe a novel transgenic model for neuroendocrine tumors, but also provide a framework for the evaluation of new, MYCN-directed therapies.

## MATERIALS AND METHODS

### Animals

Conditional expression of MYCN was achieved by crossbreeding mice carrying a floxed polyA stretch upstream of *MYCN* sequences (LSL-MYCN, [7]) with mice expressing Cre recombinase specifically in GFAP-positive cells (hGFAP-Cre, [38]). Hence, activation of MYCN and a second open reading frame coding for the luciferase gene (Fluc) in LSL-MYCN;hGFAP-Cre double transgenic animals was restricted to GFAP-positive cells expressing Cre recombinase. As the single transgenic animals derive from different strains, double transgenic animals had a mixed genetic background (FvB/ 129 SvJ x B6). Mice were genotyped for presence of transgenic cassettes and functional activity of Cre recombinase in targeted cells was proven by a “flox-out” PCR as described [7]. As the luciferase reporter (Fluc) is expressed downstream of the *MYCN* transgene, *in vivo* and *ex vivo* imaging of animals, cells and tissues was performed using a VEVO2100 device (VisualSonics). Additionally, tumor formation was monitored by abdominal palpation.

### Pathology assessment

Mice were euthanized by isoflurane and CO_2_ inhalation followed by cervical dislocation according to the local guidelines for animal handling. Necropsy was performed to macroscopically detect tumor formation. Samples of tissues were collected from multiple sites and organs and were either snap frozen or fixed in 4% paraformaldehyde for further analyses.

### Analyses of human PanNETs

Tissues sections from human PanNETs were evaluated by at least two independent pathologists to confirm the diagnosis taken from medical records. Use of patient material for the purpose of this study was approved by the Ethics committee at University of Duisburg-Essen. Macrodissection of formalin-fixed paraffin embedded (FFPE) histology blocks was performed to separately harvest tumor and surrounding normal tissue for subsequent RNA analyses. RNA was extracted using a RNeasy FFPE kit (QIAGEN, Hilden, Germany) using a previously described protocol [39]. Specific primers for amplification of the target gene, *MYCN*, by quantitative RT-PCR were designed to generate short amplicons accounting for partial degradation of RNA in FFPE samples. Results were normalised to the expression of β-Actin and tumors were evaluated as *MYCN*-positive if normalised *MYCN* expression was > 5 % than that of the positive control (a neuroblastoma cell line with high *MYCN* expression) and if the corresponding normal tissue obtained from the same FFPE sample was negative for *MYCN* gene expression.

### Western blotting, immunohistochemistry and glucagon ELISA

Organs and tumors were either snap frozen or fixed in 4% paraformaldehyde for at least 24 hours. Detection of MYCN in tumors was achieved using Western Blot analysis. In brief, tissue slices were lysed on ice in RIPA buffer (50mM HEPES, 10mM NaCl, 1% NP-40, 1% TritonX-100 and 0.1% SDS) supplemented with protease inhibitors (Roche). Separation of proteins, transfer to membranes and subsequent incubation with anti-MYCN antibody (#9405, 1:1000, Cell Signaling) was performed as described [7]. For histology, 4 μm sections were cut from paraffin blocks and stained with hematoxylin and eosin (H&E), Ki 67 (#ab16667, 1:100, Abcam), cleaved Caspase-3 (5A1E, 1:100, Cell Signaling, 9664S), Glucagon (#NCL-GLUCp, Novocastra) and Chromogranin A (#ab 15160, Abcam). Photomicrographs were recorded using a NanoZoomer 2.0HT (Hamamatsu Photonics Deutschland GmH, Herrsching, Germany). Glucagon levels in the serum of tumor-bearing or tumor-free animals were determined using a commercially available glucagon-specific ELISA (Mercodia, Uppsala, Sweden).

### mRNA expression profiling and pathway analyses

Total RNA was isolated from primary murine pancreatic and pituitary tumors, processed and hybridized to Affymetrix MG-430_2.0 oligonucleotide microarrays according to the manufacturer's protocol. Normalization of array data and identification of differentially expressed genes was achieved using Partek Genomics Suite (Partek, Michigan, MI). Furthermore, global gene expression analysis was performed using the R platform (version 3.1.1,http://www.r-project.org) and the following packages: arrayQualityMetrics (version 3.20.0), affy (version 1.42.3), limma (version 3.20.9) and vegan (version 2.2-1). To pre-process Affymetrix arrays, RMA from the affy package was used. In addition, gene set enrichment analysis (GSEA) was run on curated MSigDB gene sets and pathway activity scores calculated according to [40] using the MYCN signatures defined in [7] (and references herein). Briefly, genes were ranked for each sample and genes contained in the signature were summed for each tumor. The rank sums were divided by the average overall rank sum and log2 transformed. Student's t-test was used to determine significant differences between the pathway activity scores for different tumor groups.

### Establishing cell lines from tumors of LSL-MYCN;hGFAP-Cre mice

Pancreatic tumors were minced manually with scissors and incubated in Liberase DL (Roche) at 37°C for 30 minutes. After centrifugation, tumor cells in the supernatant were maintained in DMEM medium, 10% FCS, antibiotics (1%), L-Glutamin (1%) and sodium pyruvate (1%) for 24 hours. Long-term cultivation of tumor cells was feasible and resulted in a cell line designated Pank4. Single cell clones were generated from these cells by limited dilution. Two stable cell clones were termed IC11 and IIID2, which had differentiated and undifferentiated morphology, respectively.

### Indirect MYCN inhibition by JQ1 and MLN 8237 *in vitro* and *in vivo*

Pank4 cells were seeded into 96-well plates and treated with JQ1 and MLN 8237 for 72 hours. Metabolic activity was analysed with 3-[4,5-dimethylthiazol-3-yl]-2,5-diphenyltetrazolium bromide (MTT) assay, and used as surrogate for the number of living cells. For *in vivo* experiments, six-week old female athymic NCR (nu/nu) mice were inoculated subcutaneously with 4×10^6^ Pank4 tumor cells that were suspended in 250 μl Matrigel (Becton Dickinson, Heidelberg, Germany). Tumor growth was controlled daily and tumor volume was calculated using the formula (breadth x length x height)/2. *In vivo* luciferase imaging for tumor visualization was performed as described previously [7]. Once that tumor volume exceeded 300 mm^3^, mice were randomly assigned to one of three groups and were treated daily with either MLN 8237 (30 mg per kg body weight), JQ1 (50 mg per kg body weight) or vehicle control (12.5% DMSO in glucose). MLN 8237 was administered orally, JQ1 and the vehicle control (DMSO) via intraperitoneal injection. The animals were sacrificed once the tumor reached a volume of 1500 mm^3^. For evaluation of on target efficacy, animals were treated twice daily for three days, once the tumor volume exceeded 100 mm^3^. These animals were sacrificed four hours following the last treatment, and tumor tissue was divided and either snap-frozen in liquid nitrogen and stored at −80°C, or fixed in formalin and paraffin-embedded for immunohistochemistry.

### Statistical analyses

Statistical analyses were performed using Graph Pad Prism 5.0 (Graph Pad Software Inc., San Diego, CA, USA). Overall survival was visualized through Kaplan-Meier analyses. Mean relative proliferation was calculated by Ki-67 expression and mean relative apoptosis by positive staining of cleaved caspase 3, in transplanted LSL-MYCN;hGFAP-Cre cell lines from control, JQ1- and MLN 8237- treated groups of mice, using three representative images of each tumor. Significance was calculated by Student's t-test (*, *p* < 0.05; **, *p* < 0.01; ***, *p* < 0.001).

## SUPPLEMENTARY MATERIALS FIGURES AND TABLES






