# Supplementary material for: Characterization of pancreatic glucagon-producing tumors and pituitary gland tumors in transgenic mice overexpressing MYCN in hGFAP-positive cells
Source: Oncotarget. 2016 Oct 19;7(46):74415–26. doi: 10.18632/oncotarget.12766 (PMC5342675; doi:10.18632/oncotarget.12766)
Supplement: Supplementary file 1 [file oncotarget-07-74415-s001.pdf]

Characterization of pancreatic glucagon-producing tumors and pituitary gland tumors in transgenic mice overexpressing *MYCN* in *hGFAP*-positive cells

Supplementary Material

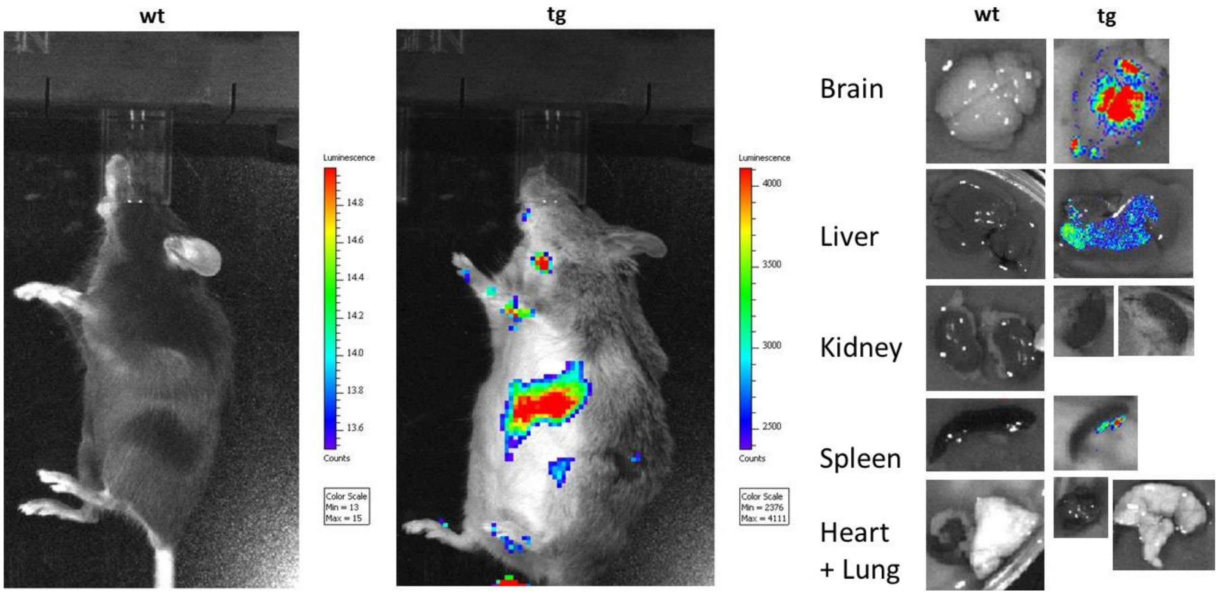

Supplemental Figure 1: Luciferase imaging of organs from wild type and LSL-MYCN;hGFAP-Cre transgenic animals.

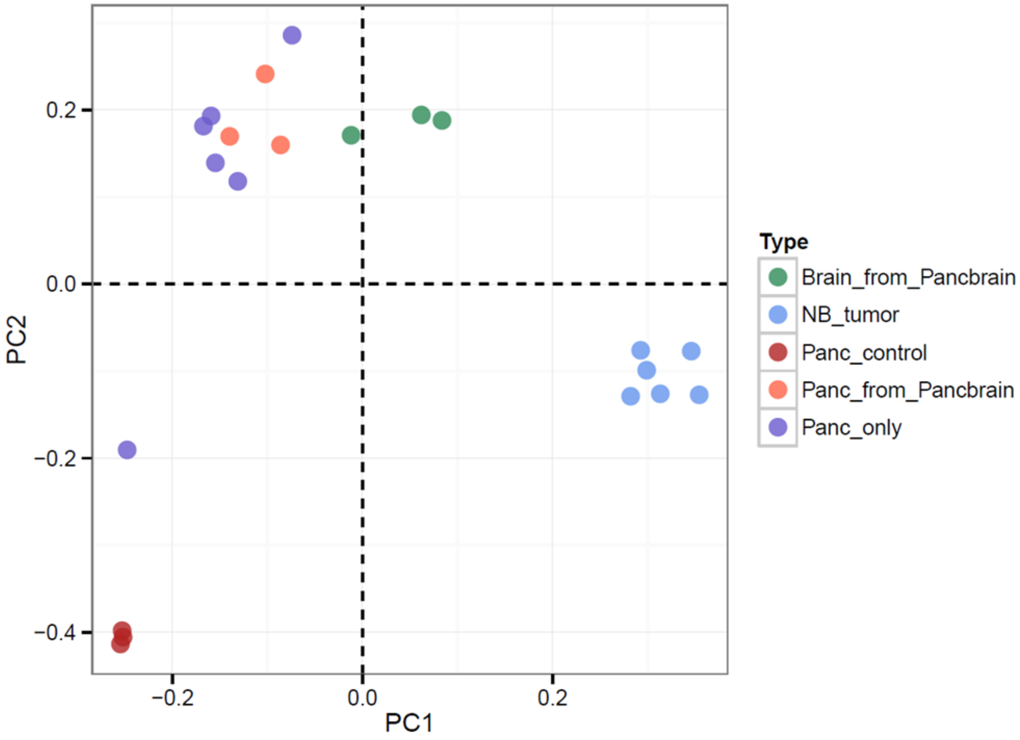

Supplemental Figure 2: Principal component analyses (PCA) revealing that all tumors derived from LSL-MYCN;hGFAP-Cre double transgenic animals group apart from controls and from an independent neuroblastoma cohort, in which LSL-MYCN induced tumors are driven by expression of DBH-iCre.

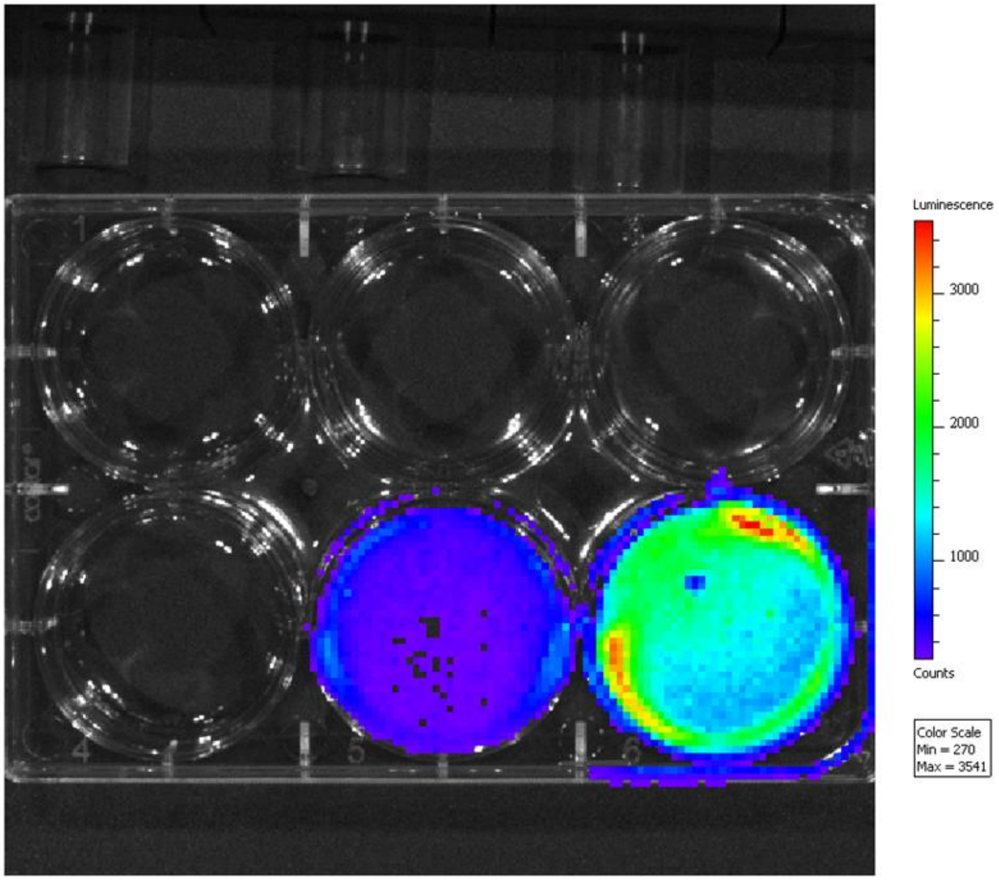

Pank 4 luciferase imaging
upper row and lower left:
control cells

Supplemental Figure 3: Luciferase imaging of Pank4 cells derived from a pancreatic tumor (lower right and middle). Upper row and lower left: Pank4 cells without addition of luciferin and control cells without expression of luciferase, respectively.

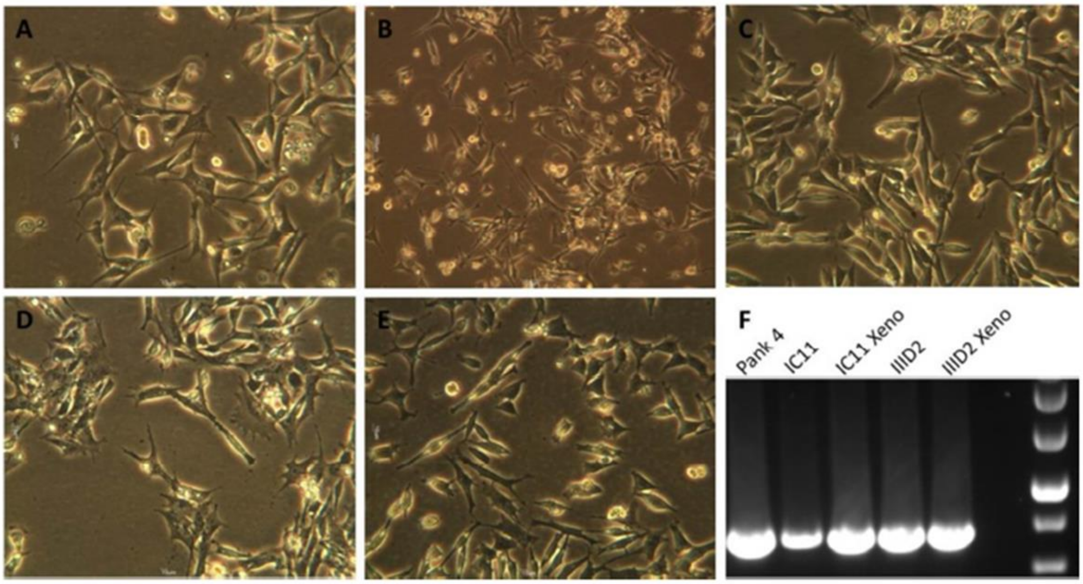

Supplemental Figure 4: A-E. Morphology of cell lines established from a LSL-MYCN;hGFAP-Cre induced tumor. **F.** PCR-based detection of polyA-stretch removal between loxP-sites in the cell lines depicted in A-E.

Supplemental Table 1: List of genes that are differentially expressed between pancreatic tumors of LSL-MYCN;hGFAP-Cre mice and normal mouse pancreas. “Probeset ID” depicts the Affymetrix ID, the gene and database symbols refer to the respective NCBI databases. In the columns labelled “P-value”, “Ratio” and “Fold change” the values for differential expression between pancreatic tumors and pancreatic normal tissue are given.
