# Supplementary material for: Characterization of pancreatic glucagon-producing tumors and pituitary gland tumors in transgenic mice overexpressing MYCN in hGFAP-positive cells
Source: Oncotarget. 2016 Oct 19;7(46):74415–26. doi: 10.18632/oncotarget.12766 (PMC5342675; doi:10.18632/oncotarget.12766)
Supplement: Supplementary file 2 [file oncotarget-07-74415-s002.docx]

| Suppl. Tab. 1 |  |  |  |  |  |
| --- | --- | --- | --- | --- | --- |
| Probeset ID | Entrez Gene | Gene Symbol | RefSeq Transcript ID | p-value(tumor vs. control) | Ratio(tumor vs. control) |
| 1449313_at | 16622 | Klk1b5 | NM_008456 | 1.69E-14 | 0.00181418 |
| 1418111_at | 67366 | Tmed11 | NM_026109 | 1.22E-12 | 0.0023771 |
| 1427380_at | 18050 | Klk1b3 | NM_008693 | 2.31E-12 | 0.0105443 |
| 1421956_at | 19668 | Rbpjl | NM_009036 | 3.27E-12 | 0.0284402 |
| 1430027_at | 66280 | 1810028F09Rik | --- | 4.37E-12 | 0.00088365 |
| 1427167_at | 100503043 | Armcx4 | NM_001202500 | 4.85E-12 | 23.7221 |
| 1453870_at | 69166 | 1810018F18Rik | NR_038140 | 5.14E-12 | 0.0174602 |
| 1460622_x_at | 216156 | Wdr18 | NM_175450 | 2.82E-11 | 0.0163607 |
| 1419314_at | 26944 | Tinag | NM_012033 | 2.99E-11 | 0.0238721 |
| 1448437_a_at | 56055 | Gtpbp2 | NM_001145979 /// NM_019581 | 3.12E-11 | 0.127936 |
| 1431742_at | 69857 | 1810053B23Rik | NR_040486 /// NR_040487 /// NR_040488 /// NR_040489 /// NR_040490 /// NR_040491 | 6.00E-11 | 0.0138108 |
| 1429523_a_at | 72002 | Slc39a5 | NM_001136237 /// NM_028051 /// NM_028092 | 6.47E-11 | 0.0204048 |
| 1448595_a_at | 19716 | Bex1 | NM_009052 | 8.89E-11 | 29.2744 |
| 1419613_at | 12836 | Col7a1 | NM_007738 | 1.21E-10 | 0.0633682 |
| 1432209_at | 74169 | 1810010K12Rik | --- | 1.70E-10 | 0.0113903 |
| 1434464_at | 208760 | Aqp12 | NM_001159658 /// NM_177587 | 1.95E-10 | 0.0280492 |
| 1429794_a_at | 18436 | P2rx1 | NM_008771 | 1.95E-10 | 0.0462186 |
| 1418785_at | 60597 | Mapk8ip2 | NM_021921 | 2.10E-10 | 7.91966 |
| 1452326_at | 69047 | Atp2c2 | NM_026922 | 3.59E-10 | 0.0385346 |
| 1448833_at | 14235 | Foxm1 | NM_008021 | 3.64E-10 | 0.054088 |
| 1434747_at | 76701 | Ctrc | NM_001033875 | 3.73E-10 | 0.00538095 |
| 1421498_a_at | 68355 | 2010204K13Rik | NR_027924 /// NR_027925 | 4.06E-10 | 20.3556 |
| 1424674_at | 106957 | Slc39a6 | NM_139143 | 4.14E-10 | 17.5322 |
| 1448628_at | 20255 | Scg3 | NM_001164790 /// NM_009130 | 4.69E-10 | 49.3262 |
| 1421372_at | 18048 | Klk1b4 | NM_010915 | 5.18E-10 | 0.0248476 |
| 1443789_x_at | 75483 | Cox8c | NM_001039049 | 5.25E-10 | 0.0593704 |
| 1424455_at | 67298 | Gprasp1 | NM_001004359 /// NM_001005385 /// NM_026081 | 5.52E-10 | 21.0842 |
| 1416364_at | 15516 | Hsp90ab1 | NM_008302 | 5.93E-10 | 4.06668 |
| 1449199_at | 17829 | Muc1 | NM_013605 | 5.98E-10 | 0.0190199 |
| 1417679_at | 14581 | Gfi1 | NM_001267621 /// NM_010278 | 7.95E-10 | 0.0602973 |
| 1435064_a_at | 57394 | Tmem27 | NM_020626 | 9.13E-10 | 58.295 |
| 1417616_at | 20446 | St6galnac2 | NM_009180 | 9.66E-10 | 0.102248 |
| 1457983_s_at | 192174 | Rwdd4a | NM_203507 | 1.03E-09 | 11.1978 |
| 1423188_a_at | 101314 | 6720456B07Rik | NM_133937 | 1.06E-09 | 5.82243 |
| 1448211_at | 76252 | Atp6v0e2 | NM_133764 | 1.14E-09 | 31.2934 |
| 1448409_at | 16970 | Lrmp | NM_008511 | 1.25E-09 | 0.0568294 |
| 1456335_at | 226866 | Gm106 | NM_001033288 | 1.88E-09 | 0.190444 |
| 1423150_at | 20394 | Scg5 | NM_009162 | 2.36E-09 | 66.5518 |
| 1420085_at | 14175 | Fgf4 | NM_010202 | 2.66E-09 | 0.240996 |
| 1418483_a_at | 14594 | Ggta1 | NM_001145821 /// NM_010283 | 2.68E-09 | 0.0354981 |
| 1448519_at | 21677 | Tead2 | NM_011565 | 2.70E-09 | 0.0306402 |
| 1455451_at | 233529 | Kctd14 | NM_001010826 /// NM_001012434 /// NM_001136235 | 2.77E-09 | 0.0252025 |
| 1428512_at | 70237 | Bhlhb9 | NM_001098222 /// NM_198161 | 2.99E-09 | 17.6194 |
| 1429013_at | 78283 | Mtap7d2 | NM_001081124 | 3.34E-09 | 62.7887 |
| 1425182_x_at | 13646 /// 13648 | Klk1b22 /// Klk1b9 | NM_010114 /// NM_010116 | 3.37E-09 | 0.00916157 |
| 1434703_at | 54616 | Extl3 | NM_018788 | 3.65E-09 | 0.280797 |
| 1453469_at | 70378 | 1810073G21Rik | --- | 3.80E-09 | 0.0254731 |
| 1460167_at | 110695 | Aldh7a1 | NM_001127338 /// NM_138600 | 3.86E-09 | 0.126815 |
| 1448555_at | 71919 | Rpap3 | NM_028003 | 3.96E-09 | 6.8486 |
| 1430352_at | 101401 | Adamts9 | NM_175314 | 4.26E-09 | 0.0841507 |
| 1428842_a_at | 12070 | Ngfrap1 | NM_001110233 /// NM_001110234 /// NM_009750 | 4.63E-09 | 8.41194 |
| 1460081_at | 54525 | Syt7 | NM_018801 /// NM_173067 /// NM_173068 | 4.70E-09 | 33.7817 |
| 1436094_at | 381677 | Vgf | NM_001039385 | 4.90E-09 | 67.3429 |
| 1426883_at | 107375 | Slc25a45 | NM_134154 | 5.09E-09 | 0.157008 |
| 1432556_a_at | 75429 | Fam183b | NM_001162878 /// NM_029283 | 5.73E-09 | 73.9852 |
| 1428501_at | 228356 | 1110051M20Rik | NM_175123 | 5.75E-09 | 5.84218 |
| 1437800_at | 171211 | Edaradd | NM_133643 | 5.94E-09 | 0.0873311 |
| 1450961_a_at | 331532 /// 594844 /// 66104 | Tceal3 /// Tceal5 /// Tceal6 | NM_001029978 /// NM_025355 /// NM_177919 | 6.23E-09 | 31.6879 |
| 1454650_at | 66854 | Trim35 | NM_029979 | 6.26E-09 | 10.7337 |
| 1455074_at | 66793 | Efcab1 | NM_025769 | 6.39E-09 | 36.7769 |
| 1425152_s_at | 67931 | Serpini2 | NM_026460 | 6.45E-09 | 0.00123607 |
| 1456846_at | 382639 | Zbtb42 | NM_001100460 | 6.56E-09 | 0.159014 |
| 1433906_at | 74438 | Clvs1 | NM_028940 | 6.60E-09 | 38.072 |
| 1418929_at | 73916 | Ift57 | NM_028680 | 7.14E-09 | 4.91576 |
| 1452899_at | 75745 | Rian | NR_028261 | 7.23E-09 | 26.5188 |
| 1418863_at | 14463 | Gata4 | NM_008092 | 7.83E-09 | 0.167565 |
| 1423626_at | 13518 | Dst | NM_133833 /// NM_134448 | 8.29E-09 | 18.7023 |
| 1422848_a_at | 54196 | Pabpn1 | NM_019402 | 8.43E-09 | 3.14372 |
| 1430211_at | 73863 | 4930415O20Rik | NM_001201322 | 8.44E-09 | 0.0664175 |
| 1423605_a_at | 17246 | Mdm2 | NM_010786 | 8.60E-09 | 5.18345 |
| 1436065_at | 12769 | Ccr9 | NM_001166625 /// NM_009913 | 8.83E-09 | 0.308888 |
| 1427025_at | 54384 | Mtmr7 | NM_001040699 /// NM_019433 | 9.09E-09 | 9.03508 |
| 1424935_at | 228858 | Gdap1l1 | NM_144891 | 9.26E-09 | 22.6424 |
| 1420597_a_at | 29866 | Cabp2 | NM_001160252 /// NM_001160253 /// NM_013878 | 9.44E-09 | 0.0245702 |
| 1437942_x_at | 71924 | Tube1 | NM_028006 | 9.72E-09 | 0.046824 |
| 1419424_at | 19213 | Ptf1a | NM_018809 | 9.87E-09 | 0.0077632 |
| 1456086_x_at | 54633 | Pqbp1 | NM_001252528 /// NM_001252529 /// NM_019478 | 1.04E-08 | 9.18708 |
| 1451442_at | 216618 | Ccdc104 | NM_025740 | 1.12E-08 | 6.26518 |
| 1436849_x_at | 14387 | Gaa | NM_001159324 /// NM_008064 | 1.13E-08 | 11.2069 |
| 1417044_at | 30949 | Lcmt1 | NM_025304 | 1.13E-08 | 0.111105 |
| 1435193_at | 319278 | A230050P20Rik | NM_175687 | 1.19E-08 | 5.67025 |
| 1454622_at | 209837 | Slc38a5 | NM_172479 | 1.21E-08 | 0.00809372 |
| 1448928_at | 15185 | Hdac6 | NM_001130416 /// NM_010413 | 1.23E-08 | 7.47562 |
| 1434641_x_at | 233878 | Sez6l2 | NM_001252566 /// NM_001252567 /// NM_144926 | 1.28E-08 | 73.9789 |
| 1437868_at | 212943 | Fam46a | NM_001160378 /// NM_001160379 | 1.32E-08 | 52.0215 |
| 1428656_at | 100045148 /// 14000 | Drosha /// LOC100045148 | NM_001130149 /// NM_026799 /// XM_001473756 | 1.38E-08 | 6.54914 |
| 1415856_at | 13723 | Emb | NM_010330 | 1.48E-08 | 38.8242 |
| 1415885_at | 12653 | Chgb | NM_007694 | 1.49E-08 | 99.0886 |
| 1451008_at | 20451 | St8sia3 | NM_009182 | 1.52E-08 | 23.2199 |
| 1425187_at | 20338 | Sel1l | NM_001039089 /// NM_011344 | 1.54E-08 | 0.0801437 |
| 1449244_at | 12558 | Cdh2 | NM_007664 | 1.57E-08 | 50.6507 |
| 1426761_at | 99982 | Kdm1a | NM_133872 | 1.77E-08 | 7.43813 |
| 1435683_a_at | 27416 | Abcc5 | NM_013790 /// NM_176839 | 1.90E-08 | 0.262035 |
| 1435021_at | 14402 | Gabrb3 | NM_001038701 /// NM_008071 | 1.93E-08 | 65.0895 |
| 1455454_at | 432720 | Akr1c19 | NM_001013785 | 1.94E-08 | 31.261 |
| 1415799_at | 60321 | Wbp11 | NM_021714 | 2.00E-08 | 3.77411 |
| 1425987_a_at | 16531 | Kcnma1 | NM_001253358 /// NM_001253359 /// NM_001253360 /// NM_001253361 /// NM_001253362 /// NM | 2.02E-08 | 7.60202 |
| 1425518_at | 56508 | Rapgef4 | NM_001204165 /// NM_001204166 /// NM_001204167 /// NM_019688 | 2.11E-08 | 19.7505 |
| 1435404_at | 214240 | Disp2 | NM_170593 | 2.11E-08 | 33.7664 |
| 1433695_at | 380686 | Cnrip1 | NM_029861 | 2.18E-08 | 19.917 |
| 1455085_at | 74284 | 1700086L19Rik | NR_030733 /// NR_030734 /// NR_030735 | 2.19E-08 | 70.2293 |
| 1440156_s_at | 269389 | Tox2 | NM_001098799 | 2.26E-08 | 39.8983 |
| 1434470_at | 80976 | Syt13 | NM_030725 | 2.32E-08 | 46.9328 |
| 1422449_s_at | 26611 | Rcn2 | NM_011992 | 2.34E-08 | 6.03386 |
| 1427044_a_at | 218038 | Amph | NM_175007 | 2.37E-08 | 39.9498 |
| 1417954_at | 20604 | Sst | NM_009215 | 2.45E-08 | 43.8481 |
| 1426260_a_at | 100048662 /// 100048744 /// 22236 /// 394430 /// 394432 /// 394433 /// 394434 /// 39443 | LOC100048662 /// LOC100048744 /// Ugt1a1 /// Ugt1a10 /// Ugt1a2 /// Ugt1a5 /// Ugt1a6a /// Ugt1a6b /// Ugt1a7c /// Ugt1a9 | NM_013701 /// NM_145079 /// NM_201410 /// NM_201641 /// NM_201642 /// NM_201643 /// NM_ | 2.46E-08 | 31.493 |
| 1418444_a_at | 56209 | Gde1 | NM_019580 | 2.46E-08 | 6.84253 |
| 1431561_a_at | 71723 | Dhx34 | NM_027883 /// XM_003689403 | 2.46E-08 | 0.0794268 |
| 1451666_at | 104112 | Acly | NM_001199296 /// NM_134037 | 2.56E-08 | 4.72803 |
| 1415837_at | 16612 | Klk1 | NM_010639 | 2.57E-08 | 0.000762528 |
| 1427851_x_at | 16019 /// 16061 /// 619916 | Igh-VJ558 /// Ighm /// Ighv1-72 | --- | 2.60E-08 | 0.104547 |
| 1433536_at | 237253 | Lrp11 | NM_172784 | 2.71E-08 | 38.7986 |
| 1417388_at | 12069 | Bex2 | NM_009749 | 2.75E-08 | 12.853 |
| 1416009_at | 56434 | Tspan3 | NM_019793 | 2.80E-08 | 4.42183 |
| 1452740_at | 77579 | Myh10 | NM_175260 | 2.83E-08 | 4.57711 |
| 1460492_at | 77547 | 8030498B09Rik | --- | 2.87E-08 | 0.239758 |
| 1433673_at | 432582 | E130309D14Rik | NM_001013784 | 2.87E-08 | 7.16417 |
| 1439604_at | 271127 | Adamts16 | NM_172053 | 3.04E-08 | 0.225423 |
| 1430913_at | 69187 | Erp27 | NM_026983 | 3.19E-08 | 0.281858 |
| 1450693_at | 56533 | Rgs17 | NM_001161822 /// NM_019958 | 3.28E-08 | 16.0424 |
| 1417176_at | 27373 | Csnk1e | NM_013767 | 3.40E-08 | 10.3303 |
| 1434073_at | 100504991 /// 245607 | Gprasp2 /// LOC100504991 | NM_001163015 /// NM_001163016 /// NM_001163017 /// XR_108220 /// XR_108221 | 3.46E-08 | 42.2017 |
| 1422445_at | 16403 | Itga6 | NM_008397 | 3.65E-08 | 0.119333 |
| 1450659_at | 24012 | Rgs7 | NM_001199003 /// NM_011880 | 3.76E-08 | 13.0273 |
| 1454668_at | 69116 | Ubr4 | NM_001160319 | 3.77E-08 | 12.0795 |
| 1451939_a_at | 51795 | Srpx | NM_016911 | 3.89E-08 | 0.201417 |
| 1423274_at | 18130 | Ints6 | NM_008715 | 4.06E-08 | 9.74594 |
| 1450252_at | 15379 | Onecut1 | NM_008262 | 4.30E-08 | 0.253097 |
| 1417535_at | 66822 | Fbxo25 | NM_025785 | 4.40E-08 | 7.51721 |
| 1425898_x_at | 229759 | Olfm3 | NM_153157 /// NM_153458 | 4.41E-08 | 67.4756 |
| 1424675_at | 106957 | Slc39a6 | NM_139143 | 4.51E-08 | 5.78312 |
| 1456653_a_at | 270685 | Mthfd1l | NM_001170785 /// NM_001170786 /// NM_172308 | 4.57E-08 | 7.73963 |
| 1460051_at | 212733 | Ccdc64b | NM_153784 | 4.61E-08 | 0.220991 |
| 1416237_at | 14012 | Mpzl2 | NM_007962 | 4.68E-08 | 0.253498 |
| 1425881_at | 114871 | Psg28 | NM_054063 | 4.72E-08 | 0.190256 |
| 1417077_at | 12033 | Bcap29 | NM_001164090 /// NM_007530 | 4.73E-08 | 6.78019 |
| 1434875_a_at | 94353 | Hmgn3 | NM_026122 /// NM_175074 | 4.75E-08 | 20.9875 |
| 1415844_at | 20983 | Syt4 | NM_009308 | 4.93E-08 | 99.1232 |
| 1431771_a_at | 65099 | Irak1bp1 | NM_001168240 /// NM_022986 | 4.97E-08 | 14.3176 |
| 1425400_a_at | 56222 | Cited4 | NM_019563 | 5.13E-08 | 0.25572 |
| 1428333_at | 76219 /// 76976 | Arxes1 /// Arxes2 | NM_029541 /// NM_029823 /// NR_003641 /// NR_003642 | 5.20E-08 | 46.2987 |
| 1424847_at | 380684 | Nefh | NM_010904 | 5.25E-08 | 15.4091 |
| 1418263_at | 30959 | Ddx25 | NM_013932 | 5.26E-08 | 13.4925 |
| 1426939_at | 69499 | Tsr2 | NM_001164578 /// NM_175146 /// NR_028392 | 5.29E-08 | 5.65585 |
| 1451329_at | 68323 | Nudt22 | NM_026675 | 5.46E-08 | 0.208104 |
| 1449463_at | 16624 | Klk1b8 | NM_008457 | 5.48E-08 | 0.0418495 |
| 1450028_a_at | 71835 | Lancl2 | NM_133737 | 5.64E-08 | 3.89943 |
| 1418067_at | 12632 | Cfl2 | NM_007688 | 5.83E-08 | 5.18537 |
| 1434989_at | 329178 | Unc80 | NM_175510 | 5.85E-08 | 19.7087 |
| 1450540_x_at | 50774 | Krtap5-1 | NM_015808 | 6.03E-08 | 0.124897 |
| 1452939_a_at | 71795 | Pitpnc1 | NM_145823 | 6.51E-08 | 6.41539 |
| 1455700_at | 74238 | Mterfd3 | NM_028832 | 6.72E-08 | 3.92177 |
| 1449318_at | 103768 | Tubg2 | NM_134028 | 6.85E-08 | 10.7639 |
| 1424942_a_at | 17869 | Myc | NM_001177352 /// NM_001177353 /// NM_001177354 /// NM_010849 | 7.23E-08 | 0.0758275 |
| 1460325_at | 80912 | Pum1 | NM_001159603 /// NM_001159604 /// NM_001159605 /// NM_001159606 /// NM_030722 | 7.49E-08 | 5.15622 |
| 1416635_at | 57319 | Smpdl3a | NM_020561 | 7.87E-08 | 0.235855 |
| 1433617_s_at | 56336 | B4galt5 | NM_019835 | 8.27E-08 | 7.90911 |
| 1439036_a_at | 11931 | Atp1b1 | NM_009721 | 8.35E-08 | 7.30316 |
| 1426225_at | 19662 | Rbp4 | NM_001159487 /// NM_011255 | 8.48E-08 | 23.797 |
| 1424572_a_at | 26914 | H2afy | NM_001159513 /// NM_001159514 /// NM_001159515 /// NM_012015 | 8.51E-08 | 5.71097 |
| 1429227_x_at | 53605 | Nap1l1 | NM_001146707 /// NM_015781 | 8.60E-08 | 4.77785 |
| 1455224_at | 72713 | Angptl1 | NM_028333 | 8.61E-08 | 0.0568843 |
| 1452032_at | 19084 | Prkar1a | NM_021880 | 8.67E-08 | 5.66042 |
| 1422208_a_at | 14697 | Gnb5 | NM_010313 /// NM_138719 | 8.83E-08 | 5.83321 |
| 1427542_at | 109205 | Sobp | NM_175407 | 8.83E-08 | 4.04342 |
| 1448737_at | 21912 | Tspan7 | NM_019634 | 8.97E-08 | 11.8177 |
| 1458591_at | 242505 | Rasef | NM_001017427 | 9.28E-08 | 0.0808476 |
| 1434897_a_at | 11739 | Slc25a4 | NM_007450 | 9.40E-08 | 4.48044 |
| 1434865_a_at | 53413 | Exoc7 | NM_001162872 /// NM_016857 | 9.86E-08 | 3.33459 |
| 1449183_at | 12846 | Comt | NM_001111062 /// NM_001111063 /// NM_007744 | 9.94E-08 | 6.72708 |
| 1452373_at | 76719 | Kansl1 | NM_001081045 | 9.95E-08 | 3.08221 |
| 1426999_at | 75553 | Zc3h14 | NM_001008506 /// NM_001160107 /// NM_001160108 /// NM_029334 /// NR_027648 | 1.01E-07 | 3.15587 |
| 1415779_s_at | 11465 | Actg1 | NM_009609 | 1.04E-07 | 3.2067 |
| 1439607_at | 241919 | Slc7a14 | NM_172861 | 1.04E-07 | 55.1452 |
| 1455148_at | 243339 | Tmem130 | NM_177735 | 1.05E-07 | 101.789 |
| 1452090_a_at | 229759 | Olfm3 | NM_153157 /// NM_153458 | 1.05E-07 | 52.6442 |
| 1439459_x_at | 104112 | Acly | NM_001199296 /// NM_134037 | 1.08E-07 | 4.75551 |
| 1422946_a_at | 13433 | Dnmt1 | NM_001199431 /// NM_001199432 /// NM_001199433 /// NM_010066 | 1.13E-07 | 4.45811 |
| 1456169_at | 226654 | Tstd1 | NM_001164525 | 1.15E-07 | 0.097437 |
| 1434326_x_at | 235431 | Coro2b | NM_175484 | 1.15E-07 | 17.7187 |
| 1451365_at | 74111 | Rbm19 | NM_028762 | 1.17E-07 | 0.194062 |
| 1460346_at | 11883 | Arsa | NM_009713 | 1.19E-07 | 3.10225 |
| 1420505_a_at | 20910 | Stxbp1 | NM_001113569 /// NM_009295 | 1.21E-07 | 49.5613 |
| 1416410_at | 18476 | Pafah1b3 | NM_008776 | 1.21E-07 | 8.55283 |
| 1417227_at | 72039 | Mccc1 | NM_023644 | 1.23E-07 | 0.210061 |
| 1423451_at | 53328 | Pgrmc1 | NM_016783 | 1.24E-07 | 3.32032 |
| 1451257_at | 216739 | Acsl6 | NM_001033597 /// NM_001033598 /// NM_001033599 /// NM_144823 | 1.25E-07 | 26.2338 |
| 1422546_at | 16201 | Ilf3 | NM_001042707 /// NM_001042708 /// NM_001042709 /// NM_010561 | 1.27E-07 | 3.34255 |
| 1455765_a_at | 20927 | Abcc8 | NM_011510 | 1.27E-07 | 62.8416 |
| 1451544_at | 213233 | Tapbpl | NM_145391 | 1.28E-07 | 4.77374 |
| 1444970_at | 97241 | C79870 | --- | 1.30E-07 | 0.288479 |
| 1435519_at | 215449 | Rap1b | NM_024457 | 1.35E-07 | 3.17577 |
| 1423753_at | 68010 | Bambi | NM_026505 | 1.36E-07 | 25.0436 |
| 1439327_at | 320924 | Ccbe1 | NM_178793 | 1.40E-07 | 0.165781 |
| 1418942_at | 67694 | Ift74 | NM_026319 | 1.41E-07 | 5.04621 |
| 1417630_at | 17346 | Mknk1 | NM_021461 | 1.42E-07 | 0.132343 |
| 1451272_a_at | 67921 | Ube2f | NM_026454 | 1.45E-07 | 0.182016 |
| 1424009_at | 30053 | Reg3d | NM_001161741 /// NM_013893 | 1.47E-07 | 0.00730966 |
| 1418244_at | 67877 | Naa20 | NM_001141965 /// NM_026425 | 1.47E-07 | 7.32793 |
| 1449992_at | 114662 | Prss29 | NM_053260 | 1.48E-07 | 0.273741 |
| 1428540_at | 77574 | Fam115a | NM_029930 | 1.49E-07 | 12.4999 |
| 1416418_at | 57436 | Gabarapl1 | NM_020590 | 1.49E-07 | 9.41736 |
| 1424145_at | 75210 | Prr3 | NM_001165892 /// NM_145487 /// NR_028507 /// NR_028516 | 1.52E-07 | 4.35733 |
| 1416863_at | 64296 | Abhd8 | NM_022419 | 1.56E-07 | 6.63039 |
| 1429402_at | 74782 | Glt8d2 | NM_029102 | 1.56E-07 | 0.24038 |
| 1460629_at | 94092 | Trim16 | NM_053169 | 1.58E-07 | 0.157762 |
| 1434403_at | 114716 | Spred2 | NM_033523 | 1.59E-07 | 5.66723 |
| 1455502_at | 228355 | Madd | NM_001177719 /// NM_001177720 /// NM_001177721 /// NM_001177722 /// NM_001177723 /// NM | 1.59E-07 | 4.59982 |
| 1416805_at | 68659 | Fam198b | NM_133187 | 1.68E-07 | 0.130113 |
| 1426571_at | 101772 | Ano1 | NM_001242349 /// NM_178642 | 1.72E-07 | 0.205522 |
| 1452787_a_at | 15469 | Prmt1 | NM_001252476 /// NM_001252477 /// NM_019830 /// NR_045521 | 1.78E-07 | 4.53024 |
| 1452666_a_at | 68875 | Tmcc2 | NM_178874 | 1.79E-07 | 22.8116 |
| 1451070_at | 14567 | Gdi1 | NM_010273 | 1.80E-07 | 8.94 |
| 1418691_at | 19739 | Rgs9 | NM_001165934 /// NM_011268 | 1.83E-07 | 14.4016 |
| 1447837_x_at | 80905 | Polh | NM_030715 | 1.84E-07 | 0.189947 |
| 1427108_at | 213673 | 9530068E07Rik | NM_153117 | 1.84E-07 | 6.00066 |
| 1418213_at | 94179 | Krt23 | NM_033373 | 1.90E-07 | 0.106323 |
| 1451958_at | 16061 /// 636067 | Igh-VJ558 /// LOC636067 | XM_003689439 | 1.92E-07 | 0.297963 |
| 1449706_s_at | 26424 | Nr5a2 | NM_001159769 /// NM_030676 | 1.92E-07 | 0.151399 |
| 1427019_at | 19283 | Ptprz1 | NM_001081306 /// NM_011219 /// NM_178180 | 1.94E-07 | 75.6026 |
| 1431303_at | 106504 | Stk38 | NM_134115 | 1.95E-07 | 0.27485 |
| 1423653_at | 11928 | Atp1a1 | NM_144900 | 1.97E-07 | 3.19993 |
| 1453586_at | 12495 | Entpd1 | NM_009848 | 1.97E-07 | 0.0810635 |
| 1450397_at | 17755 | Mtap1b | NM_008634 | 1.97E-07 | 23.8251 |
| 1437338_x_at | 74195 | Elp3 | NM_001253812 /// NM_028811 /// NR_045599 | 2.00E-07 | 4.07555 |
| 1422448_at | 21785 | Tff2 | NM_009363 | 2.03E-07 | 0.00500376 |
| 1451534_at | 214189 | Scgn | NM_145399 | 2.03E-07 | 20.9874 |
| 1428415_at | 66381 | Rnf113a2 | NM_025525 | 2.04E-07 | 4.64748 |
| 1421391_at | 22355 | Vipr2 | NM_009511 | 2.05E-07 | 0.313385 |
| 1450848_at | 65111 | Dap3 | NM_001164533 /// NM_022994 | 2.07E-07 | 3.69641 |
| 1451588_at | 69123 | Eci3 | NM_026947 | 2.18E-07 | 0.0831107 |
| 1456076_at | 246700 | Defb19 | NM_145157 | 2.22E-07 | 0.328272 |
| 1437472_at | 382018 | Unc13a | NM_001029873 | 2.31E-07 | 8.89225 |
| 1448530_at | 66355 | Gmpr | NM_025508 | 2.31E-07 | 16.8563 |
| 1450380_at | 105298 | Epdr1 | NM_134065 | 2.33E-07 | 9.36677 |
| 1427943_at | 75572 | Acyp2 | NM_029344 | 2.35E-07 | 6.86176 |
| 1415780_a_at | 67416 | Armcx2 | NM_001166397 /// NM_001166398 /// NM_026139 | 2.35E-07 | 5.93792 |
| 1416828_at | 20614 | Snap25 | NM_011428 | 2.41E-07 | 84.0841 |
| 1419754_at | 17918 | Myo5a | NM_010864 | 2.43E-07 | 10.4537 |
| 1445546_at | 106267 | AI844685 | --- | 2.43E-07 | 0.021064 |
| 1449509_at | 20365 | Serf1 | NM_011353 | 2.48E-07 | 0.260531 |
| 1449271_a_at | 56016 | Hebp2 | NM_019487 | 2.48E-07 | 3.58186 |
| 1415770_at | 83669 | Wdr6 | NM_031392 | 2.50E-07 | 20.3947 |
| 1429958_x_at | 68977 | Haghl | NM_026897 | 2.53E-07 | 7.01929 |
| 1451453_at | 13143 | Dapk2 | NM_010019 | 2.54E-07 | 0.181156 |
| 1451288_s_at | 67884 | 1810043G02Rik | NM_026431 | 2.55E-07 | 6.96217 |
| 1436030_at | 320508 | Cachd1 | NM_198037 | 2.55E-07 | 0.103421 |
| 1438288_x_at | 68786 | 1110059G02Rik | --- | 2.59E-07 | 0.0665223 |
| 1444128_at | 71302 | Arhgap26 | NM_175164 | 2.61E-07 | 0.0896935 |
| 1417179_at | 56224 | Tspan5 | NM_019571 | 2.68E-07 | 5.07439 |
| 1454764_s_at | 105727 | Slc38a1 | NM_001166456 /// NM_001166458 /// NM_134086 | 2.79E-07 | 11.5701 |
| 1458666_at | 51897 | Atg13 | NM_145528 | 2.79E-07 | 0.301765 |
| 1424306_at | 83603 | Elovl4 | NM_001145974 /// NM_148941 | 2.81E-07 | 23.0345 |
| 1450660_at | 19286 | Pts | NM_011220 | 2.88E-07 | 7.38589 |
| 1448363_at | 22601 | Yap1 | NM_001171147 /// NM_009534 | 2.89E-07 | 0.096243 |
| 1430095_at | 216393 | D930020B18Rik | NM_177335 | 2.93E-07 | 0.330922 |
| 1453091_s_at | 68614 | Letmd1 | NM_134093 | 2.93E-07 | 6.28298 |
| 1426804_at | 20586 | Smarca4 | NM_001174078 /// NM_001174079 /// NM_011417 | 2.94E-07 | 3.2325 |
| 1427229_at | 15357 | Hmgcr | NM_008255 | 2.95E-07 | 6.37066 |
| 1434274_at | 105689 | Mycbp2 | NM_207215 | 2.98E-07 | 4.18364 |
| 1434376_at | 12505 | Cd44 | NM_001039150 /// NM_001039151 /// NM_001177785 /// NM_001177786 /// NM_001177787 /// NM | 3.05E-07 | 24.0837 |
| 1437851_x_at | 19826 | Rnps1 | NM_001080127 /// NM_001080128 /// NM_009070 | 3.08E-07 | 3.1267 |
| 1453279_x_at | 77055 | Krt76 | NM_001033177 | 3.09E-07 | 0.209235 |
| 1433756_at | 74648 | S100pbp | NM_029036 | 3.17E-07 | 7.87723 |
| 1421470_at | 14829 | Grpr | NM_008177 | 3.23E-07 | 0.253388 |
| 1437627_at | 237400 | Mex3d | NM_198615 | 3.42E-07 | 4.8338 |
| 1436150_at | 71901 | 2310028H24Rik | NM_001159583 /// NM_027993 | 3.45E-07 | 6.16996 |
| 1415949_at | 12876 | Cpe | NM_013494 | 3.46E-07 | 3.71483 |
| 1432416_a_at | 100046628 /// 18148 | LOC100046628 /// Npm1 | NM_001252260 /// NM_001252261 /// NM_008722 /// XM_001476529 | 3.48E-07 | 3.82371 |
| 1423891_at | 103140 | Gstt3 | NM_133994 | 3.64E-07 | 0.144945 |
| 1433977_at | 54710 | Hs3st3b1 | NM_018805 | 3.66E-07 | 0.195747 |
| 1426449_a_at | 18744 | Pja1 | NM_001083110 /// NM_008853 | 3.67E-07 | 5.11811 |
| 1434034_at | 223753 | Cerk | NM_145475 | 3.68E-07 | 16.2392 |
| 1451421_a_at | 66049 | Rogdi | NM_133185 | 3.70E-07 | 4.15753 |
| 1431176_at | 66371 | Chmp4c | NM_025519 | 3.74E-07 | 0.165482 |
| 1445297_at | 207227 | Stxbp5l | NM_001114611 /// NM_001114612 /// NM_001114613 /// NM_172440 | 3.80E-07 | 22.5765 |
| 1426010_a_at | 13823 | Epb4.1l3 | NM_013813 | 3.80E-07 | 13.5654 |
| 1425350_a_at | 17876 | Myef2 | NM_001162417 /// NM_001162418 /// NM_010852 | 3.80E-07 | 8.02792 |
| 1430046_at | 73162 | Otud3 | NM_028453 | 3.80E-07 | 0.264721 |
| 1419615_at | 64177 | Trpv6 | NM_022413 | 3.84E-07 | 0.154339 |
| 1452686_s_at | 52174 | Tmem222 | NM_025667 | 3.87E-07 | 4.3788 |
| 1428221_at | 78267 | Klhdc8b | NM_030075 | 3.88E-07 | 8.54249 |
| 1423555_a_at | 99899 | Ifi44 | NM_133871 | 3.89E-07 | 0.249304 |
| 1455865_at | 53626 | Insm1 | NM_016889 | 3.89E-07 | 111.287 |
| 1460565_at | 98396 | Slc41a1 | NM_173865 | 3.92E-07 | 0.109601 |
| 1423835_at | 218820 | Zfp503 | NM_145459 | 3.96E-07 | 0.0815153 |
| 1448485_at | 14598 | Ggt1 | NM_008116 | 4.00E-07 | 0.0109539 |
| 1453558_at | 75040 | Efcab10 | NM_029152 | 4.04E-07 | 18.6185 |
| 1459987_s_at | 12462 | Cct3 | NM_001243065 /// NM_009836 | 4.09E-07 | 6.15192 |
| 1438653_x_at | 54138 | Atxn10 | NM_016843 | 4.11E-07 | 3.29241 |
| 1440285_at | 243725 | Ppp1r9a | NM_181595 | 4.13E-07 | 7.26261 |
| 1438370_x_at | 100503659 | Dos | NM_001195268 /// NM_015761 /// NR_036582 | 4.17E-07 | 17.5537 |
| 1448727_at | 114606 | Tle6 | NM_053254 | 4.18E-07 | 0.176029 |
| 1451508_at | 214048 | Larp1b | NM_001040399 /// NM_027663 /// NM_145988 | 4.22E-07 | 0.17979 |
| 1452237_at | 15463 | Agfg1 | NM_010472 | 4.32E-07 | 8.3058 |
| 1455927_x_at | 67711 | Nsmce1 | NM_026330 | 4.34E-07 | 3.52273 |
| 1438396_at | 320634 | Ocrl | NM_177215 | 4.42E-07 | 14.1251 |
| 1441603_at | 20607 | Sstr3 | NM_009218 | 4.47E-07 | 28.9056 |
| 1425030_at | 52521 | Zfp622 | NM_144523 | 4.59E-07 | 5.09163 |
| 1420855_at | 13717 | Eln | NM_007925 | 4.71E-07 | 0.14643 |
| 1420770_at | 16617 | Klk1b24 | NM_010643 | 4.72E-07 | 0.0686803 |
| 1437395_at | 230594 | Zcchc11 | NM_175472 | 4.82E-07 | 6.62176 |
| 1460549_a_at | 52563 | Cdc23 | NM_178347 | 4.86E-07 | 7.10355 |
| 1432560_at | 16604 | Klk1b7-ps | NR_033120 | 4.88E-07 | 0.238565 |
| 1436503_at | 232400 | BC048546 | NM_001001179 | 4.91E-07 | 50.1035 |
| 1453016_at | 72938 | Hspb11 | NM_028394 | 4.91E-07 | 5.69001 |
| 1422320_x_at | 18690 | Phxr5 | NM_008836 /// XR_035720 /// XR_035721 /// XR_035722 | 4.91E-07 | 0.264112 |
| 1423983_at | 233878 | Sez6l2 | NM_001252566 /// NM_001252567 /// NM_144926 | 4.94E-07 | 56.683 |
| 1453120_at | 52837 | Tmx4 | NM_029148 | 4.96E-07 | 18.2315 |
| 1418034_at | 69527 | Mrps9 | NM_023514 | 5.00E-07 | 3.00778 |
| 1435138_at | 270028 | Fam155a | NM_173446 | 5.10E-07 | 57.0173 |
| 1425760_a_at | 18739 | Pitpnm1 | NM_001136078 /// NM_008851 | 5.17E-07 | 4.66249 |
| 1421950_at | 18637 | Pfdn2 | NM_011070 | 5.20E-07 | 4.79568 |
| 1424177_at | 74166 | Tmem38a | NM_144534 | 5.23E-07 | 8.89115 |
| 1419056_at | 20167 | Rtn2 | NM_001025364 /// NM_013648 | 5.25E-07 | 14.8429 |
| 1453317_a_at | 13992 | Khdrbs3 | NM_010158 | 5.26E-07 | 46.5756 |
| 1424524_at | 71712 | Dram1 | NM_027878 | 5.26E-07 | 0.0834673 |
| 1439014_at | 239796 | 1600021P15Rik | NM_177718 | 5.36E-07 | 4.20337 |
| 1436814_at | 100040049 | 1810010D01Rik | NR_033626 | 5.39E-07 | 0.0609127 |
| 1450683_at | 56370 | Tagln3 | NM_019754 | 5.41E-07 | 4.79876 |
| 1450940_at | 14545 | Gdap1 | NM_010267 | 5.41E-07 | 21.6925 |
| 1437185_s_at | 19240 | Tmsb10 | NM_001039392 /// NM_001190327 /// NM_025284 | 5.53E-07 | 3.9857 |
| 1422758_at | 54371 | Chst2 | NM_018763 | 5.62E-07 | 0.0260205 |
| 1436815_x_at | 100040049 | 1810010D01Rik | NR_033626 | 5.70E-07 | 0.0989299 |
| 1455802_x_at | 23795 | Agr2 | NM_011783 | 5.84E-07 | 0.302663 |
| 1418780_at | 56050 | Cyp39a1 | NM_018887 | 5.86E-07 | 0.0337131 |
| 1419650_at | 22763 | Zfr | NM_011767 | 5.90E-07 | 3.52765 |
| 1424221_at | 96935 | Susd4 | NM_144796 | 6.01E-07 | 5.89245 |
| 1423319_at | 15242 | Hhex | NM_008245 | 6.03E-07 | 25.1905 |
| 1445457_at | 230828 | Il22ra1 | NM_178257 | 6.04E-07 | 0.189297 |
| 1416481_s_at | 56295 | Higd1a | NM_019814 | 6.10E-07 | 0.124278 |
| 1433707_at | 14397 | Gabra4 | NM_010251 | 6.16E-07 | 0.017363 |
| 1460403_at | 101739 | Psip1 | NM_133948 | 6.20E-07 | 6.10026 |
| 1458331_x_at | 67935 | Ces5a | NM_001003951 | 6.25E-07 | 0.329102 |
| 1429455_at | 66691 | Gapvd1 | NM_025709 | 6.33E-07 | 3.50173 |
| 1422634_a_at | 57276 | Vsig2 | NM_020518 | 6.34E-07 | 0.199547 |
| 1451205_at | 19172 | Psmb4 | NM_008945 | 6.47E-07 | 3.01365 |
| 1451356_at | 66471 | Anp32e | NM_001253757 /// NM_001253758 /// NM_023210 | 6.52E-07 | 3.13826 |
| 1416067_at | 15982 | Ifrd1 | NM_013562 | 6.54E-07 | 0.0972128 |
| 1419163_s_at | 100037258 | Dnajc3 | NM_008929 | 6.59E-07 | 0.152124 |
| 1451405_at | 110821 | Pcca | NM_144844 | 6.82E-07 | 0.306082 |
| 1440286_at | 214931 | Fbxl16 | NM_001164225 | 6.87E-07 | 14.4268 |
| 1423893_x_at | 11785 | Apbb1 | NM_001253885 /// NM_001253886 /// NM_001253887 /// NM_009685 | 6.88E-07 | 6.66412 |
| 1421756_a_at | 14760 | Gpr19 | NM_001167693 /// NM_001167694 /// NM_001167695 /// NM_001167696 /// NM_001167697 /// NM | 6.94E-07 | 10.0183 |
| 1418178_at | 226180 | Ina | NM_146100 | 6.95E-07 | 26.3669 |
| 1451218_at | 192193 | Edem1 | NM_138677 | 6.99E-07 | 0.0872899 |
| 1436470_at | 116838 | Rims2 | NM_001256382 /// NM_001256383 /// NM_001256384 /// NM_053271 | 7.06E-07 | 10.7489 |
| 1449932_at | 104318 | Csnk1d | NM_027874 /// NM_139059 | 7.16E-07 | 3.96235 |
| 1454984_at | 16880 | Lifr | NM_001113386 /// NM_013584 | 7.24E-07 | 0.103575 |
| 1424533_a_at | 67291 | Ccdc137 | NM_152807 | 7.29E-07 | 4.03353 |
| 1426929_at | 108013 | Celf4 | NM_001146292 /// NM_001146293 /// NM_001146294 /// NM_001146295 /// NM_001174074 /// NM | 7.30E-07 | 16.9264 |
| 1434352_at | 101148 | B630005N14Rik | NM_175312 | 7.32E-07 | 3.71347 |
| 1460292_a_at | 93761 | Smarca1 | NM_053123 | 7.45E-07 | 23.5693 |
| 1448260_at | 22223 | Uchl1 | NM_011670 | 7.45E-07 | 33.36 |
| 1457700_at | 320997 | Cyp4f39 | NM_177307 | 7.51E-07 | 0.321052 |
| 1429265_a_at | 59044 | Rnf130 | NM_021540 | 7.56E-07 | 4.26703 |
| 1424192_at | 68948 | 1500011H22Rik | NM_026883 | 7.65E-07 | 6.11773 |
| 1456074_at | 70061 | Sdr9c7 | NM_027301 | 7.68E-07 | 0.242477 |
| 1456200_at | 69718 | Ipmk | NM_027184 | 7.90E-07 | 3.78324 |
| 1453187_at | 433904 | Ociad2 | NM_026950 | 7.92E-07 | 0.113183 |
| 1452259_at | 228829 | Phf20 | NM_172674 | 7.94E-07 | 4.08588 |
| 1419027_s_at | 56356 | Gltp | NM_019821 | 7.96E-07 | 0.236936 |
| 1417943_at | 14706 | Gng4 | NM_010317 | 7.99E-07 | 38.8419 |
| 1422558_at | 14431 | Gamt | NM_010255 | 8.00E-07 | 0.0165353 |
| 1437497_a_at | 15519 | Hsp90aa1 | NM_010480 | 8.01E-07 | 3.78569 |
| 1423045_at | 68092 | Ncbp2 | NM_026554 | 8.07E-07 | 6.1078 |
| 1428116_a_at | 100040531 /// 100040563 /// 100310872 /// 21648 | Dynlt1a /// Dynlt1b /// Dynlt1c /// Dynlt1f | NM_001166627 /// NM_001166629 /// NM_001166630 /// NM_001199947 /// NM_001199948 /// NM | 8.17E-07 | 4.99726 |
| 1426957_at | 27223 | Trp53bp1 | NM_013735 | 8.18E-07 | 4.96042 |
| 1419358_at | 81840 | Sorcs2 | NM_030889 | 8.25E-07 | 11.5831 |
| 1425476_at | 12830 | Col4a5 | NM_001163155 /// NM_007736 | 8.32E-07 | 0.0896519 |
| 1455390_at | 233065 | Alkbh6 | NM_198027 | 8.34E-07 | 3.94552 |
| 1439827_at | 239337 | Adamts12 | NM_175501 | 8.62E-07 | 0.229222 |
| 1439044_at | 30944 | Zfp354c | NM_013922 | 8.67E-07 | 4.94783 |
| 1417923_at | 18481 | Pak3 | NM_001195046 /// NM_001195047 /// NM_001195048 /// NM_001195049 /// NM_008778 | 8.86E-07 | 19.6765 |
| 1439009_at | 237107 | Gnl3l | NM_001168600 /// NM_198110 | 8.98E-07 | 3.80015 |
| 1448717_at | 270076 | Gcdh | NM_001044744 /// NM_008097 | 9.06E-07 | 0.324118 |
| 1425979_a_at | 217335 | Fbf1 | NM_172571 | 9.06E-07 | 3.83473 |
| 1433596_at | 72685 | Dnajc6 | NM_001164583 /// NM_001164584 /// NM_001164585 /// NM_198412 | 9.07E-07 | 13.1108 |
| 1451489_at | 71998 | Slc25a35 | NM_028048 | 9.07E-07 | 0.0907792 |
| 1456133_x_at | 16419 | Itgb5 | NM_001145884 /// NM_010580 | 9.16E-07 | 3.88653 |
| 1426261_s_at | 100048662 /// 100048744 /// 22236 /// 394430 /// 394432 /// 394433 /// 394434 /// 39443 | LOC100048662 /// LOC100048744 /// Ugt1a1 /// Ugt1a10 /// Ugt1a2 /// Ugt1a5 /// Ugt1a6a /// Ugt1a6b /// Ugt1a7c /// Ugt1a9 | NM_013701 /// NM_145079 /// NM_201410 /// NM_201641 /// NM_201642 /// NM_201643 /// NM_ | 9.25E-07 | 34.3368 |
| 1456837_at | 233879 | Asphd1 | NM_001039645 | 9.25E-07 | 4.05531 |
| 1433452_at | 102941 | B630019K06Rik | NM_175327 /// NR_045448 | 9.31E-07 | 28.849 |
| 1428347_at | 76884 | Cyfip2 | NM_001252459 /// NM_001252460 /// NM_133769 | 9.36E-07 | 3.69999 |
| 1417216_at | 18715 | Pim2 | NM_138606 | 9.46E-07 | 5.16846 |
| 1418191_at | 24110 | Usp18 | NM_011909 | 9.51E-07 | 29.3403 |
| 1447725_at | 77469 | C030034E14Rik | --- | 9.56E-07 | 52.6298 |
| 1417680_at | 16493 | Kcna5 | NM_145983 | 9.74E-07 | 27.3681 |
| 1429151_at | 71833 | Dcaf7 | NM_027946 | 9.75E-07 | 4.91777 |
| 1449948_at | 67475 | Ero1lb | NM_026184 | 9.76E-07 | 0.194772 |
| 1448766_at | 14618 | Gjb1 | NM_008124 | 9.82E-07 | 0.0663647 |
| 1449848_at | 14675 | Gna14 | NM_008137 | 9.96E-07 | 0.0768394 |
| 1430538_at | 70123 | 2210013O21Rik | NM_027327 /// NR_028432 | 9.97E-07 | 3.93571 |
| 1447999_x_at | 100042025 /// 100048117 /// 14433 | Gapdh /// LOC100042025 /// LOC100048117 | NM_008084 /// XM_001476707 /// XM_001479371 | 9.99E-07 | 3.46206 |
| 1416626_at | 18778 | Pla2g1b | NM_011107 | 9.99E-07 | 0.00370599 |
| 1416275_at | 171429 | Slc26a6 | NM_134420 | 1.02E-06 | 0.211509 |
| 1426517_at | 14687 | Gnaz | NM_010311 | 1.03E-06 | 13.5969 |
| 1425159_at | 68338 | Golt1a | NM_026680 | 1.03E-06 | 9.18801 |
| 1448902_at | 67009 | Ttc23 | NM_001168475 /// NM_001168476 /// NM_001168477 /// NM_025905 | 1.04E-06 | 0.201855 |
| 1460341_at | 226971 | Plekhb2 | NM_145516 /// NM_175421 | 1.04E-06 | 19.2391 |
| 1428622_at | 100505173 /// 97998 | Deptor /// LOC100505173 | NM_001037937 /// NM_145470 /// XR_106933 | 1.05E-06 | 0.317777 |
| 1451359_at | 210992 | Lpcat1 | NM_145376 | 1.07E-06 | 6.45356 |
| 1418123_at | 22248 | Unc119 | NM_011676 | 1.07E-06 | 6.77834 |
| 1436210_at | 235533 | Gk5 | NM_177352 | 1.07E-06 | 0.222827 |
| 1419268_at | 23795 | Agr2 | NM_011783 | 1.08E-06 | 0.100544 |
| 1424234_s_at | 17286 | Meox2 | NM_008584 | 1.08E-06 | 0.261346 |
| 1416523_at | 19752 | Rnase1 | NM_011271 | 1.09E-06 | 0.00274541 |
| 1423952_a_at | 110310 | Krt7 | NM_033073 | 1.10E-06 | 43.731 |
| 1435258_at | 51875 | Tmem141 | NM_001040130 /// NM_001109993 | 1.12E-06 | 7.50912 |
| 1438659_x_at | 66098 | Chchd6 | NM_001167736 /// NM_025351 | 1.12E-06 | 14.4817 |
| 1435652_a_at | 14678 | Gnai2 | NM_008138 | 1.12E-06 | 4.76106 |
| 1422836_at | 171170 | Mbnl3 | NM_134163 | 1.12E-06 | 0.13407 |
| 1456584_x_at | 236539 | Phgdh | NM_016966 | 1.13E-06 | 0.113341 |
| 1448649_at | 13809 | Enpep | NM_007934 | 1.13E-06 | 22.898 |
| 1455293_at | 235497 | Leo1 | NM_001006122 /// NM_001039522 | 1.14E-06 | 3.40901 |
| 1450052_at | 16563 | Kif2a | NM_001145779 /// NM_008442 | 1.15E-06 | 4.70406 |
| 1450658_at | 23794 | Adamts5 | NM_011782 | 1.16E-06 | 0.286704 |
| 1460250_at | 66042 | Sostdc1 | NM_025312 | 1.16E-06 | 0.0334052 |
| 1418525_at | 18536 | Pcm1 | NM_023662 | 1.17E-06 | 6.19334 |
| 1436623_at | 93685 | Entpd7 | NM_053103 | 1.18E-06 | 0.101303 |
| 1453377_at | 72281 | Sh2d4a | NM_028182 | 1.18E-06 | 0.100343 |
| 1457671_at | 26557 | Homer2 | NM_001164086 /// NM_001164087 /// NM_011983 /// NM_177029 | 1.21E-06 | 0.0637358 |
| 1424376_at | 104445 | Cdc42ep1 | NM_027219 | 1.22E-06 | 0.121919 |
| 1416130_at | 19122 | Prnp | NM_011170 | 1.22E-06 | 15.7431 |
| 1438912_at | 15193 | Hdgfrp2 | NM_008233 | 1.22E-06 | 0.331883 |
| 1433876_at | 378937 | Lrrc24 | NM_198119 | 1.23E-06 | 10.1429 |
| 1438032_at | 380916 | Lrch1 | NM_001033439 /// NM_001252132 | 1.23E-06 | 3.84413 |
| 1433339_at | 77724 | 6030460B20Rik | --- | 1.24E-06 | 0.330378 |
| 1435338_at | 12571 | Cdk6 | NM_009873 | 1.24E-06 | 0.0684276 |
| 1415911_at | 16210 | Impact | NM_008378 | 1.25E-06 | 13.1827 |
| 1425293_a_at | 66995 | Zcchc18 | NM_001035509 /// NM_001035510 /// NM_025893 | 1.26E-06 | 18.3789 |
| 1416997_a_at | 15114 | Hap1 | NM_010404 /// NM_177981 | 1.27E-06 | 15.8329 |
| 1436208_at | 65247 | Asb1 | NM_001039126 /// NM_023046 | 1.27E-06 | 3.42366 |
| 1415698_at | 105348 | Golm1 | NM_001035122 /// NM_027307 | 1.28E-06 | 6.12283 |
| 1454752_at | 666794 | Rbm24 | NM_001081425 | 1.31E-06 | 10.7913 |
| 1428973_s_at | 66061 | Tctex1d2 | NM_025329 | 1.31E-06 | 4.46981 |
| 1452309_at | 68178 | Cgnl1 | NM_026599 | 1.32E-06 | 0.28757 |
| 1429896_at | 74756 | 5830408B19Rik | --- | 1.33E-06 | 9.88526 |
| 1454709_at | 100201 | Tmem64 | NM_181401 | 1.35E-06 | 0.275199 |
| 1450913_at | 56386 | B4galt6 | NM_019737 | 1.35E-06 | 7.02138 |
| 1448465_at | 18082 | Nipsnap1 | NM_008698 | 1.36E-06 | 5.55425 |
| 1446756_at | 208188 | Ghsr | NM_177330 | 1.37E-06 | 10.9146 |
| 1428211_at | 74443 | P4htm | NM_028944 | 1.39E-06 | 6.53221 |
| 1453647_at | 22317 /// 78668 | E130112N10Rik /// Vamp1 | NM_001080557 /// NM_009496 /// NR_015604 | 1.39E-06 | 0.315497 |
| 1428227_at | 19712 | Rest | NM_011263 | 1.39E-06 | 0.145932 |
| 1433870_at | 217138 | Prr15l | NM_146026 | 1.40E-06 | 0.133429 |
| 1448883_at | 19141 | Lgmn | NM_011175 | 1.41E-06 | 12.2578 |
| 1435977_at | 29877 | Hdgfrp3 | NM_013886 | 1.42E-06 | 10.8361 |
| 1440882_at | 16975 | Lrp8 | NM_001080926 /// NM_053073 /// NR_033496 | 1.42E-06 | 7.57595 |
| 1428209_at | 406217 | Bex4 | NM_212457 | 1.42E-06 | 4.92328 |
| 1460687_at | 67956 | Setd8 | NM_030241 | 1.44E-06 | 0.256116 |
| 1435879_at | 23797 | Akt3 | NM_011785 | 1.44E-06 | 8.82632 |
| 1452247_at | 14359 | Fxr1 | NM_001113188 /// NM_001113189 /// NM_008053 | 1.45E-06 | 3.48503 |
| 1451224_at | 56807 | Scamp5 | NM_020270 | 1.46E-06 | 8.8382 |
| 1428305_at | 18549 | Pcsk2 | NM_008792 | 1.48E-06 | 98.876 |
| 1450344_a_at | 19218 | Ptger3 | NM_011196 | 1.49E-06 | 0.0080185 |
| 1427270_a_at | 100383 | Bsdc1 | NM_133889 | 1.49E-06 | 3.27159 |
| 1421201_a_at | 56191 | Tro | NM_001002272 /// NM_019548 /// NM_207679 | 1.52E-06 | 10.8494 |
| 1451302_at | 68618 | 1110012L19Rik | NM_026787 | 1.53E-06 | 3.51385 |
| 1434592_at | 72472 | Slc16a10 | NM_001114332 /// NM_028247 | 1.54E-06 | 11.654 |
